# Learning millisecond protein dynamics from what is missing in NMR spectra

**DOI:** 10.1101/2025.03.19.642801

**Published:** 2026-07-01

**Authors:** Hannah K. Wayment-Steele, Gina El Nesr, Ramith Hettiarachchi, Adedolapo M. Ojoawo, Hasindu Kariyawasam, Sergey Ovchinnikov, Dorothee Kern

**Affiliations:** 1 Department of Integrated Structural and Computational Biology, Scripps Research & Howard Hughes Medical Institute, La Jolla, CA; 2 Biophysics Program, Stanford University, Stanford, CA; 3 Department of Biology, Massachusetts Institute of Technology, Cambridge, MA; 4 Current address: Ray and Stephanie Lane Computational Biology Department, School of Computer Science, Carnegie Mellon University, Pittsburgh, PA; 5 Center for Advanced Imaging, Faculty of Arts and Sciences, Harvard University, Cambridge, MA; 6 Current address: Cornell Bowers College of Computing and Information Science, Gates Hall, Ithaca, NY

## Abstract

Many proteins’ biological functions rely on interconversions between multiple conformations occurring at micro- to millisecond (μs-ms) timescales. A lack of standardized, large-scale experimental data has hindered obtaining a more predictive understanding of these motions. After curating >100 Nuclear Magnetic Resonance (NMR) relaxation datasets, we realized an observable for μs-ms dynamics might be hiding in plain sight. Millisecond dynamics can cause NMR signals to broaden beyond detection, leaving some residues not assigned in the chemical shift datasets of ~10,000 proteins deposited in the Biological Magnetic Resonance Data Bank (BMRB)^1^. We made the bold assumption that residues missing assignments are exchange-broadened due to μs-ms motions and trained various deep learning models to predict missing assignments. Strikingly, these models also predict exchange measured via NMR relaxation experiments, indicative of μs-ms dynamics. The best of these models, which we named Dyna-1, leverages an intermediate layer of the multimodal language model ESM-3^2^. Notably, dynamics directly linked to biological function, including enzyme catalysis and ligand binding, are particularly well predicted by Dyna-1, which parallels our findings that residues experiencing μs-ms exchange are more conserved. We anticipate the datasets and models presented here will be transformative in unlocking the common language of dynamics and function.

## Introduction.

**Fig. 1.**
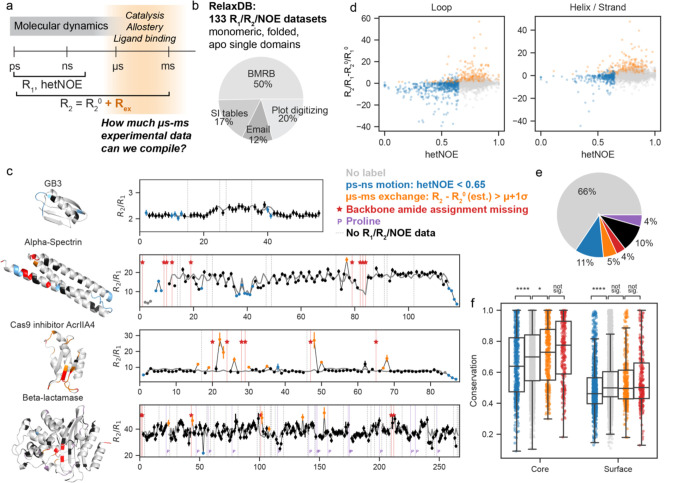
A benchmark of 133 protein NMR backbone relaxation measurements demonstrates residues with ps-ns motion are less conserved, and residues with μs-ms exchange are more conserved. (a) Many biological processes occur at a μs-ms time regime, yet experimental data in this regime have not existed at a sufficient scale for machine learning. NMR R_1_, R_2_ and heteronuclear NOE (hetNOE) measure ps to low ns motion, while millisecond timescale exchange causes increased R_2_ (R_ex_). (b) The “RelaxDB” benchmark we curated consists of 133 single-domain ^15^N protein backbone datasets with associated labels for the nature of motion present at each residue. Pie chart contains the origin of the data in RelaxDB. (c) Example raw R_2_/R_1_ data (black) and comparison to calculation from rigid tumbling using HYDRONMR (grey line)^48,49^ to obtain labels. Labels are shown in the different colors in legend and plotted onto the AF2 predicted structures. Error bars on R_2_/R_1_ data are calculated as the maximum of uncertainties reported by authors or uncertainties estimated from the data (see Methods). (d) Distribution of hetNOE vs. calculated change in R_2_/R_1_ between experiment and rigid tumbling calculation. Blue: ps-ns motion. Orange: μs-ms exchange. Grey: no label. (e) Distribution of RelaxDB labels. (f) Calculating sequence conservation for these residues demonstrates that residues with μs-ms exchange are more conserved than residues with no assigned label, which in turn are more conserved than residues with ps-ns motion. Residues with missing backbone amide assignments are no different in conservation than residues with μs-ms exchange. n=12,584 residues. Box plots depict median and 25/75% interquartile range, whiskers = 1.5 *interquartile range. Statistical comparisons by two-tailed independent-samples t-test; no multiple comparisons adjustment. *: 0.01 ≤ p ≤ 0.05; ****: p ≤ 0.0001.

The functions of proteins often depend on their ability to interconvert between multiple conformations^3^. Our ability to understand fundamental biological mechanisms would be significantly improved if we had greater ability to predict multiple conformations and the timescales at which they interconvert. Computationally modelling the motions of proteins has been a longstanding goal since the first molecular dynamics (MD) simulations of proteins^4^. However, our predictive power for protein motions pales in comparison to our predictive power for single structures. AlphaFold-2 (AF2)^5^ demonstrated that the experimental data available in the Protein Data Bank (PDB)^6^, coupled with large-scale sequencing data and modern deep-learning architectures, could achieve unprecedented accuracy in structure prediction. However, the task of predicting *protein dynamics* lacks what the task of predicting single structures had: large-scale, standardized benchmarks of experimental observables. Any computational method, be it deep-learning-based or simulation-based, currently faces a paucity of standardized experimental data available on dynamics to evaluate on, let alone train from (Fig. 1a).

We focus here on μs-ms dynamics, since it is well established that motions on this timescale are directly linked to biological function^7^ and are often concerted in nature^8^. Where can we find experimental data on μs-ms dynamics? The primary source of structural data used in deep learning, the structure information in the PDB, lacks time information. Time-resolved X-ray diffraction and cryo-electron microscopy methods are gaining increasing traction, but processing these datasets requires nontrivially deconvolving signal to identify multiple conformations. In contrast, the last 50 years of methods development in NMR has led to experiments in which the presence of μs-ms dynamics *creates* signal in the form the observable *R*_*ex*_. R_ex_ is an increase in R_2_ relaxation times that unambiguously indicates that the monitored atom is experiencing changes in its chemical environment at the μs-ms timescale^9,10^.

We wished to address the lack of experimental dynamics benchmarks by curating standardized datasets of R_ex_ measurements on backbone amides. In analyzing the resulting “RelaxDB” dataset of 133 proteins, we found that residues with μs-ms exchange were most conserved. This suggested that with more experimental data on μs-ms dynamics, it might be feasible to use an evolutionary-guided deep learning approach, such as a protein language model, to predict μs-ms dynamics. This task—namely, can a model predict the presence of a concerted, millisecond-timescale process?—is distinct from other existing tasks: predicting backbone flexibility or order parameters^11,12^ or predicting multiple conformations without information on timescales^13,14^. External conditions such as temperature, pH and cofactors will change the rate and relative populations of such a process, but not the underlying process itself.

We were curious if the ~10,000 proteins with deposited NMR chemical shift information in the BMRB could provide a useful dynamics observable for training a model. We hypothesized that missing ^15^N backbone assignments are primarily caused by exchange-broadening due to μs-ms motions. We tested this key assumption by training deep learning models to predict which residues are missing assignments, and consequently evaluating if these models also have predictive power for measured R_ex_ for residues that are assigned. Our results demonstrate that signatures of millisecond-timescale dynamics are indeed learnable by modern deep learning approaches trained with these NMR data.

The developed model, Dyna-1, is able to predict biologically relevant μs-ms exchange as assessed on proteins characterized by many independent labs. Notably, our model led to reinterpretation of published NMR relaxation data in several ways. While investigating “false negatives” in the RelaxDB dataset, we noticed that some proteins interact with phosphate ions as part of their biological function, yet the NMR relaxation experiments were conducted in phosphate buffer leading to artificially increased R_2_ due to buffer binding. Dyna-1 did not predict exchange from such non-specific phosphate binding. Conversely, Dyna-1 did predict millisecond exchange that typical analysis of Carr-Purcell-Meiboom-Gill (CPMG) experiments missed, but more careful consideration of the existing NMR data validated. We anticipate Dyna-1 as well as the datasets used for training and evaluation will be transformative in better understanding how biomolecular dynamics leads to function.

## Results

### Residues with exchange are most conserved

To curate datasets of standardized R_ex_ measurements, we started with the experiment where R_ex_ is most directly measured: Carr-Purcell-Meiboom-Gill (CPMG) relaxation dispersion^9,10,15,16^. However, we could only find fewer than 10 sets of complete ^15^N backbone CPMG data either publicly available or from private correspondences. We next turned to the more abundant “R_1_/R_2_/hetNOE” (R_1_ relaxation, R_2_ relaxation, heteronuclear nuclear Overhauser enhancement) experiments, which provide information on backbone pico-nanosecond (ps-ns) motions and μs-ms exchange. We compiled data on 163 distinct protein domains (Fig. 1b), the earliest of which were published in 1990^17^. Because R_ex_ reports on any process causing a change in chemical environment, which can include multimerization or the binding-unbinding of a ligand, we only included datasets of apo proteins with no cofactors present, where the authors had verified a monomeric sample. We also excluded intrinsically disordered proteins. We devised a framework to robustly and systematically label residues with exchange while accounting for anisotropic effects (see Methods).

Fig. 1c depicts the degree to which our framework accounts for anisotropic effects for four example proteins from RelaxDB. The protein GB3 is small yet has pronounced anisotropy in its R_2_/R_1_ arising from its helix; it was a system first used to demonstrate that anisotropic tumbling leads to heightened R_2_/R_1_^18^. This “zigzag” pattern due to differing directions of backbone N-H amide bond vectors in a helix is also evident in the highly-anisotropic helical bundle of alpha spectrin^19^. Our fitting process discerns between anisotropic data features and increases in R_2_/R_1_ due to μs-ms exchange, exemplified in AcrIIA4 Cas9 inhibitor^20^ and beta-lactamase^21^. The original 163 proteins were reduced to 133 as some proteins had extensive missing data or ps-ns motion indicative of disordered regions and could not be reliably fit with this workflow (see Methods). All datasets are in Extended Data Fig. 1.

Fig. 1d depicts the distribution of all residues from these proteins along the two experimental observables defining the presence of different timescales of motion: namely, dR_2_/R_1_ (see Methods) for μs-ms exchange, and hetNOE for ps-ns motion. As expected, many residues with low hetNOE have a dR_2_/R_1_ less than zero, as motions at the ps-ns timescale lead to both a lower hetNOE and a reduced R_2_ value. A few residues have both fast motions detected via hetNOE and elevated dR_2_/R_1,_ indicative of μs-ms exchange. If anything, we anticipate that this method of looking at elevated R_2_/R_1_ is under-estimating μs-ms exchange for residues where motions are present at more than one timescale, as μs-ms exchange in elevating R_2_ and ps-ns motion decreasing R_2_ would cancel.

Despite comprising only 133 proteins, the RelaxDB benchmark represents an unprecedented amount of experimental information on dynamics in one place. Fig. 1e depicts the overall distribution of different labels assigned per residue. With this curated dataset in hand, we were interested in seeing if there were any eminent relationships between sequence and dynamics. When calculating sequence conservation, we noticed a striking trend: the residues with μs-ms exchange were more conserved than residues with no designated label, which in turn were more conserved than residues with ps-ns motion. Overall, solvent-exposed residues are less conserved than non-solvent-exposed residues (Fig. 1f). Residues involved in μs-ms processes are often involved in functions such as allosteric signaling, catalysis, or ligand binding. Nevertheless, we were surprised that this could be quantitatively detected in as few as 133 proteins. Further analysis revealed that the predicted local distance difference test (pLDDT) measure from AF2 is lowest for residues with ps-ns motion and shows little discrepancy between residues with no motion and μs-ms exchange (Extended Data Fig. 2).

Though we hoped RelaxDB would be useful for evaluating models, any model trained with only 133 proteins is unlikely to generalize. In looking at the data, we realized that our initial curation of the data lacked a key observable that could also give information on μs-ms dynamics. Some residues in the proteins of RelaxDB could not be assigned a label because they did not have measured R_1_/R_2_/hetNOE values. These residues lacking relaxation data fall into two fundamentally different categories. In the first category, a residue is assigned, but the authors did not report R_1_/R_2_/hetNOE values for a variety of reasons: possibly the peaks were too overlapped to fit exponentials reliably or an exponential did not fit well, for instance due to weak signal intensity. In the second category, a residue is missing R_1_/R_2_/hetNOE values because the residue was unable to be assigned in the first place. Crucially, residues can be missing from chemical shift assignments if they are “exchange-broadened”, i.e., they are experiencing exchange between two states where the chemical shift difference of the two states is comparable to the frequency at which the residue is interconverting. Therefore, we identified the corresponding published assignments for each protein and created labels to distinguish whether residues without R_1_/R_2_/hetNOE data were assigned or not. We hypothesized that if the residues missing assignments were also reporting on residues with μs-ms exchange, they would show similar trends in conservation to residues with R_ex_. Indeed, sequence conservation is statistically indistinguishable between residues with R_ex_ and residues with missing assignments (Fig. 1f). Fundamentally, whether a residue is not assigned because it is exchange-broadened or can be assigned but displays R_ex_ is dependent on the chemical shift difference of its multiple states relative to their interconversion rate.

### Curating proxy labels for exchange

**Fig. 2.**
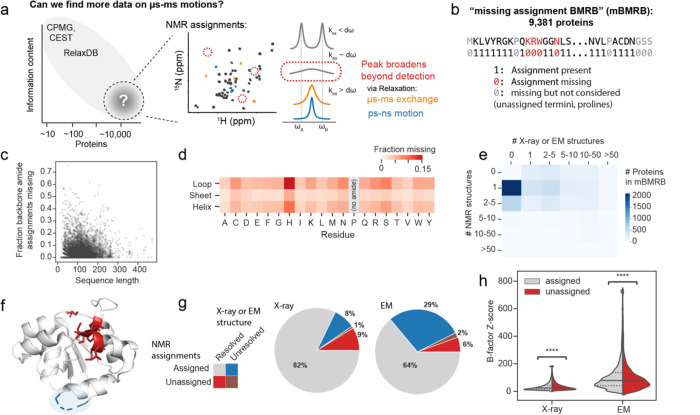
Missing NMR amide backbone assignments in BMRB used as label for μs-ms exchange. (a) In seeking more experimental data for μs-ms dynamics, we realized that data on roughly 10,000 proteins exists in the form of missing backbone amide assignments in the BMRB. Theoretical NMR line-shapes for 2-site exchange on different timescales shown. (b) Scheme for curating the “missing assignment BMRB” (mBMRB) dataset. (c) Fraction backbone amides missing vs. sequence length, not considering prolines or unassigned termini. (d) Fraction of backbone amides missing, disaggregated by amino acid and secondary structure type, in structures predicted by ESMFold^22^. (e) The majority of proteins in the BMRB have only one NMR structure and no X-ray or EM structure. (f) Residues with missing NMR peaks are distinct from residues missing in X-ray and EM structures, as exemplified by the MAP kinase binding domain of DUSP 16 (BMRB:19330, PDB:3TG3). Residues with missing assignments are in red, residues with missing X-ray density are in blue. (g) Fraction of residues assigned/unassigned in the mBMRB compared with resolved/unresolved in X-ray or EM structures shows little overlap. (h) B-factors of residues with present or missing NMR peaks have similar distributions. Solid lines: median, dotted lines: 25/75% percentile. In 2(g,h): n=71,704 protein datasets, n=306,842 residues. Statistical comparisons by two-tailed independent-samples t-test; no multiple comparisons adjustment. ****: p ≤ 0.0001.

We realized that these missing assignments were present at orders-of-magnitude higher counts than in just the relaxation measurements (Fig. 2a); the Biological Magnetic Resonance Bank (BMRB) contains ~12,000 proteins with deposited chemical shifts. Could a model trained on all the proteins in the BMRB, where the label is “is this residue assigned or not?” generalize to predicting μs-ms exchange? The key assumption such a model would be leveraging is that backbone amide assignments are primarily missing due to exchange, as opposed to incomplete assignments or other reasons. Of course, residues that are completely exchange-broadened represent a subset of all residues with exchange. If we use residue counts in RelaxDB as a rough proxy for the relative abundances of detectable R_ex_ versus missing assignments, we observe that 5% of residues have elevated R_2_/R_1_, whereas 4% have missing assignments (Fig. 1e). This rough estimate concludes that nearly half of the residues with exchange have missing assignments, which certainly is not all, but also not negligible.

We curated a new dataset: the mBMRB. In this dataset, we removed proteins that might have systematic, non-exchange reasons for assignments being missing (see Methods, Extended Data Fig. 3). This resulted in a dataset containing 9,381 proteins, almost two orders of magnitude more than in RelaxDB (Fig. 2b). This dataset comprises “positives” (for μs-ms dynamics), which are missing assignments curated as carefully as possible, but of course the “negatives” (no μs-ms dynamics) in this dataset are not unambiguously so: many residues are assignable while also experiencing exchange. Nevertheless, after applying these filters, we observed that a substantial fraction of proteins across the BMRB had significant numbers of non-proline and non-termini missing assignments (Fig. 2c). Disaggregating residues by identity and secondary structure type (calculated from structures predicted by ESMFold^22^) reveals that missing assignments span across both loops and structured elements for all amino acid types, though more frequently in loops (Fig. 2d). We note that while there will doubtlessly be noise in these labels arising from mis-assignment or other errors, there is well-established literature showing that in certain regimes of machine learning, noise can actually assist model convergence as a form of regularization^23,24^.

We next compared the information in these “missing assignment” labels to sources of heterogeneity and uncertainty present in other forms of publicly available structure data to inform on what models trained on the PDB such as AF2 may or may not have already been exposed to (see Methods). 45% of these proteins (2680/5906) had no X-ray or EM structures but had one or more NMR structures (Fig. 2e). For proteins that were characterized by X-ray and/or EM and assigned by NMR, residues with missing assignments appear distinct from residues unresolved in X-ray or EM structure models (Fig. 2f,g). Of the residues that are resolved, the distributions of B-factors are similar for both assigned and unassigned residues (Fig. 2h). In summary, our mBMRB dataset contains unique information on thousands of proteins in comparison to information present in X-ray and EM structures in the PDB.

### Predicting missing NMR assignments

**Fig. 3.**
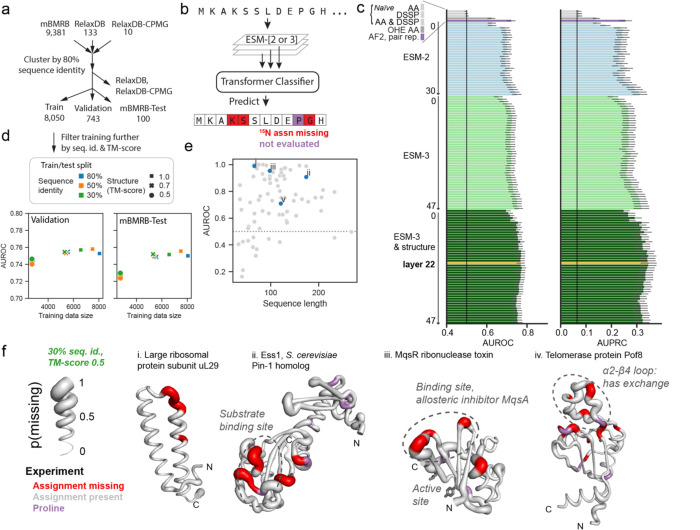
Deep learning has predictive power for the “missing assignment BMRB” (mBMRB). (a) Train/validation/test split scheme to train from the mBMRB while holding out 100 proteins from mBMRB as well as all RelaxDB and RelaxDB-CPMG data as test sets. (b) Schematic of deep learning architectures tested. (c) AUROC and AUPRC on validation set testing layers from ESM-2^22^ and ESM-3^2^ models, AlphaFold2^5^ pair representation, and baselines. AA: amino acid. DSSP: secondary structure assignment. OHE AA: one-hot encoded amino acid. Layer 22 was chosen as the best-performing model (Dyna-1). Error bars represent 95% confidence interval for AUROC and AUPRC evaluated over proteins in validation set. (d) Performance is not significantly impacted by removing sequence or structure homologues more stringently from training. (e) AUROC from Dyna-1, trained using the 30% sequence identity and 0.5 TM-score dataset split, on the mBMRB-Test set. Proteins in blue are shown in (f). (f) Examples of Dyna-1 predictions. Structures are generated with ESMFold from mBMRB sequence (BMRB entries 5977, 50787, 51503, and 50002 for i-iv, respectively), the thickness of the tube is the probability missing as predicted by Dyna-1. Residues are colored for experimental data as follows: red: missing backbone assignment, grey: assignment present, purple: proline (no data).

We next tested if current deep learning models when trained with these curated NMR data have the capacity to predict which assignments are missing, and second if such learning could transfer to predictive power for μs-ms exchange. We created a train/validation/test data split, initially holding out any sequences with >80% sequence identity to the RelaxDB or RelaxDB-CPMG datasets to use later as higher quality experimental evaluations with labels of exchange (Fig. 3a). To contextualize model performance on this new task, we began by calculating a set of naïve baselines. Of these, the best performing model on the validation set used sequence, secondary structure, and SASA (see Methods, Extended Data Fig. 4a).

We were curious to explore the predictive power of existing deep learning models on this task, since AF2^5^ and ESM-2^22^ have shown predictive capabilities beyond the tasks for which they were trained. For example, the protein language model ESM-2 was trained in the self-supervised task of masked language modeling yet has predictive power for secondary structure, 3D structure, and functional labels. In this “transfer learning” paradigm, extracted representations from pretrained models are used to train new models with different datasets on related downstream tasks^25^. We designed architectures to test structure-focused representations from AF2^5^ pair representations (“AF2, pair rep.”), sequence-only representations in ESM-2^22^, and interactions between sequence and structure by using the multimodal language model ESM-3^2^ (see Methods, Fig. 3b, Extended Data Fig. 5). We first compared these architectures by training with our curated NMR dataset (mBMRB) at 80% sequence identity cutoff; after assessing the models’ predictive capability on this novel task given a majority of the available data, we then retrained models with a more stringently filtered training set (see below).

We observed that all three of these pretrained models out-performed other baseline models (Fig. 3c). The best-performing model was layer 22 of ESM-3 if provided both sequence and structure models as input, with an AUROC of 0.77. Intriguingly, the last layer of ESM-2 model achieved an AUROC of 0.75, and the AF2-pair model, which did not use MSAs, and rather only structure input, achieved 0.71. This suggests that both sequence and structure information provide utility in this new task. Performance from all ESM-2 and ESM-3 layers tested are depicted in Fig. 3c. AUPRC (Fig. 3c, right) mirrors AUROC values. We selected based on AUPRC since it is slightly more discriminative than AUROC. AUPRC visualizations for all subsequent per-protein comparisons discussed are included in Extended Data Fig. 6.

We tested the effect of sequence and structure similarity in the training data on performance by training with more stringent training splits: we tested 50% and 30% sequence identity cutoffs and structure-based similarity cutoffs of TM-score at 0.5 and 0.7. These significantly reduced the size of the training set, with the smallest including only ~2700 proteins. The most stringent training split decreased the AUROC of Dyna-1 on the validation set to 0.74 (Fig. 3d), and comparably on the remaining evaluation datasets in this work (Extended Data Fig. 6). We did not observe any individual proteins with significant jumps in AUROC between our most stringent training set (30% sequence identity, 0.5 TM-score cutoff) and our most lenient (80% sequence identity, 1.0 TM-score cutoff), which would have indicated model memorization (Extended Data Fig. 5d). We note that model performance does not improve significantly when given almost three-fold more data (cf. Extended Data Fig. 6). The final model leverages the embeddings of layer 22 of ESM-3, uses both sequence and structure as inputs, and was trained with the most stringent data split (30% sequence identity, 0.5 TM-score cutoff).

Fig. 3e depicts AUROC per protein for those with one or more missing assignments in the mBMRB-Test dataset. Fig. 3f depicts the structures of some of the best-performing proteins in the test set with tube sized by the output of Dyna-1, i.e., the predicted probability each residue is missing, “p(missing)”, in comparison to residues with missing assignments (red). The missing assignments in example (iii) are related to the function of this protein; in ribonuclease toxin MqsR, loop β2-β3 is known to have exchange, and its flexibility is reported to be related to its ability to bind mRNA^26^. The antitoxin MqsA acts on this protein not by directly inhibiting its active site, but rather by sequestering the β2-β3 loop and prohibiting its motion, which allows the ribonuclease to show broad non-specificity towards a variety of mRNA substrates^26^.

Though these results appeared promising, missing assignments are an incomplete indicator of μs-ms dynamics, since residues can experience exchange broadening (R_ex_) that does not necessarily preclude their assignment. Hence, “false positives” in this data cannot be held as ground truth. This is exemplified in (iv), the telomerase protein Pof8^27^. Dyna-1 predicts high p(missing) for the α2-β4 loop, yet most of these residues are assigned. However, the authors described that assigning the α2-β4 loop and analyzing subsequent relaxation data for the loop was difficult “due to severe line broadening”^27^, a clear indicator of μs-ms exchange. We therefore needed to evaluate Dyna-1’s predictions using the more-informative relaxation data in RelaxDB to gain a better sense of the model’s predictive capability for μs-ms dynamics. This next evaluation also tests if model learning can “transfer” beyond only predicting residues that are exchange-broadened to the extent that precludes their assignment to residues that are assignable, but have exchange detectable via relaxation.

### Dyna-1 predicts μs-ms exchange

**Fig. 4.**
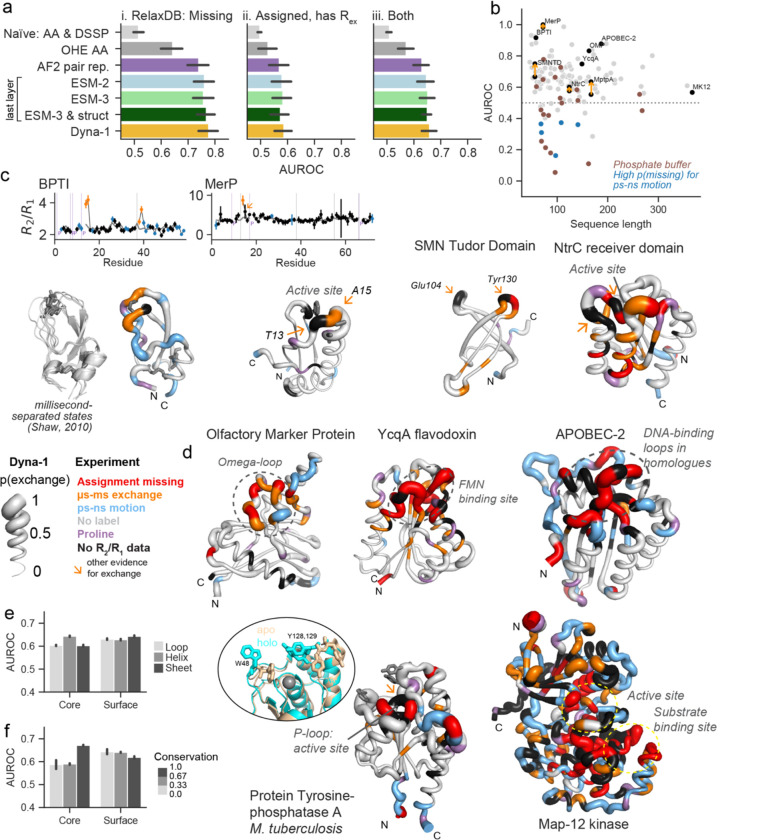
Dyna-1 predicts biologically-relevant micro-millisecond dynamics in NMR relaxation data. (a) Representative models evaluated on (i) missing assignments in RelaxDB, (ii) residues with assigned R_ex_, and (iii) both missing and R_ex_. Error bars represent 95% confidence interval for mean evaluated over proteins in RelaxDB. Error bars represent 95% confidence interval for mean AUROC evaluated over proteins in RelaxDB (n=112). (b) AUROC per protein vs. sequence length for Dyna-1 in RelaxDB for both missing assignments and residues with R_ex_. Only shown are proteins with 2 or more residues with missing assignment or exchange. Brown: proteins with exchange from phosphate buffer. Blue: proteins with high p(exchange) primarily in regions with ps-ns motions (cf. Extended Data Fig. 7). Orange arrows: proteins where follow-up on residues with no relaxation data resulted in increased AUROC (see c,d). (c) Dyna-1 predictions on smaller proteins offer an opportunity for detailed comparisons to experimental data. BPTI agrees with experimental R_2_/R_1_ data^31^ (top) and heterogeneity among kinetically-distinct substates in molecular dynamics^32^ (left). Mercuric Transport Protein, SMN Tudor Domain, and NtrC receiver domain offer prospective tests of residues with high p(exchange) for which no data was available (black), but other evidence for R_ex_ (orange arrow). R_2_/R_1_ error bars are from the original curated datasets. (d) Representative Dyna-1 predictions for larger proteins. Biologically-relevant features are annotated in grey. The Dyna-1 prediction for MptpA, a phosphatase from *M. tuberculosis*, recapitulates measurements from NMR as well as structural heterogeneity in sidechains regulating specificity (W48, Y128, Y129) (apo: 2LUO, holo: 1U2P). (e) AUROC for residues binned by core and surface designation and secondary structure type indicates that Dyna-1 has the most predictive power in core helices and surface loops. (f) AUROC binned by core/surface location and sequence conservation indicate that Dyna-1 has the most predictive power for more conserved residues. In (e,f), bars represent mean; error bars represent standard error evaluated via bootstrapping (n = 13,810 residues).

We found that when evaluating only missing assignments (Fig. 4ai), representative models achieved comparable performance to the mBMRB validation and test sets. To test if the model indeed generalized beyond the data with which it was trained, we held out missing assignments and asked how well it could predict which assigned residues have exchange (Fig. 4aii). Performance does decrease across all models compared to the task of predicting missing assignments, but AF2-pair, ESM-2, and ESM-3-based models achieved AUROC well above the controls, indicating that the models can generalize learning from predicting missing assignments (e.g. p(missing)) to predicting exchange within assigned residues (e.g. p(exchange)). To evaluate the performance of the model to predict μs-ms exchange as manifested in both forms, we consider both missing assignments and residues with measured μs-ms exchange in RelaxDB (Fig. 4aiii). Out of the representative models, layer 22 of ESM-3 given both sequence and structure again performed the best in this evaluation, same as in the mBMRB validation set. We named this final model “Dyna-1”.

Fig. 4b depicts AUROC for all proteins in RelaxDB vs. sequence length. We first noticed that there were numerous proteins in RelaxDB with an AUROC of <0.5, i.e. worse than a random predictor. By carefully reading the corresponding NMR papers, we learned that some of these were DNA- or RNA-binding proteins measured in phosphate buffer, and that the unpredicted but experimentally reported exchange often occurred at positively-charged surface residues (Extended Data Fig. 7). Several publications directly state this phenomena as an artefact about either their own or others’ datasets being a limitation to interpreting measured exchange in the system they investigate.^28,29^ We annotated these low-performing datasets further and found that out of the 22 proteins with AUROC ≤ 0.5, 10 of these (45%) are proteins that during their biological function would be expected to interact with phosphate groups (i.e. DNA- or RNA-binding proteins or signaling domains), yet were experimentally characterized in phosphate buffer. Removing these proteins from RelaxDB increased Dyna-1’s overall performance from an AUROC of 0.63 to 0.66.

Figs. 4c,e depict representative proteins where structures are sized by Dyna-1 predicted p(exchange) and colored by experimental data for comparison. In Bovine Pancreatic Trypsin Inhibitor (BPTI), Cys14, Lys15, Cys38, and Arg39 are known to undergo millisecond transitions from disulfide bond isomerization.^30,31^ Promisingly for our automated labeling scheme, these four residues are also labeled as having μs-ms exchange from relaxation data in ref. ^31^ (depicted in Fig. 4c, top). Shaw et al. identified 5 kinetically distinct states on the micro-millisecond timescales via molecular dynamics simulations^32^ (depicted in Fig. 4c, left). Strikingly, Dyna-1 predicts high p(exchange) for the two loops containing these residues (Fig. 4c, right), to achieve an AUROC of 0.92 for predicting millisecond exchange in BPTI. While investigating Dyna-1 predictions in RelaxDB and revisiting original datasets, we found several residues with no relaxation data yet other evidence exists for exchange, underscoring Dyna-1’s predictive power (see supporting information, orange arrows in Fig. 4c,d).

In assessing what motifs Dyna-1 was able to predict in larger proteins (Fig. 4d), we were intrigued to see that many of the dynamic motifs it correctly predicts also have biological relevance. In flavodoxin YcqA, Dyna-1 predicts high exchange in the loop and helical regions that correspond to the FMN ligand-binding site. In Olfactory Marker Protein from humans, Dyna-1 predicts high p(exchange) in the omega-loop, which is described in ref. ^33^ to have substantial R_ex_ and is highly conserved. More recent studies indicate that the omega loop serves as a nuclear export signal^34^. In APOBEC-2, Dyna-1 predicts high p(exchange) in a region that in structure homologues is known to control the specificity of DNA substrates^35^. For Tyrosine-phosphatase A from *M. tuberculosis* (MptpA)^36^, Dyna-1 has high p(exchange) in loops containing residues with differing orientations between the apo and holo structures (apo: 2LUO, holo: 1U2P), and which are postulated to be involved in regulating enzyme specificity^36^. To test our hypothesis that Dyna-1 was performing better in predicting conserved motions, we calculated AUROC across different sets of residues categorized by secondary structure, conservation score, and core and surface designation. Indeed, we found that Dyna-1 had the most predictive power for more conserved residues in the core, supporting our observations on individual proteins (Fig. 4e,f).

Dyna-1 predictions in the MAP kinase p38 gamma (MK12) are indicative of room for future improvement. For MK12, we used experimental labels derived from the authors’ model-free fitting to multiple field strengths^37^. MK12 is a highly dynamic protein with ps-ns and μs-ms processes occurring throughout its structure, and p(exchange) predicted by Dyna-1 was high across the whole protein. Improving discrimination between ps-ns motions and μs-ms exchange is the subject of future work. We identified numerous proteins where Dyna-1’s highest p(exchange) is in regions with ps-ns motion (blue in Fig. 4b, structures in Extended Data Fig. 7c). One challenge in interpreting these is that residues can experience both ps-ns motions and μs-ms exchange, and single field strength measurements for R1 and R2 are insufficient to experimentally quantify motions at two timescales simultaneously. We expect that if dynamics is present at more than one timescale, because μs-ms exchange elevates R_2_ and ps-ns motion decreases R_2_, our current experimental labels for those likely underestimate R_ex_.

### Dyna-1 reveals overlooked exchange

**Fig. 5.**
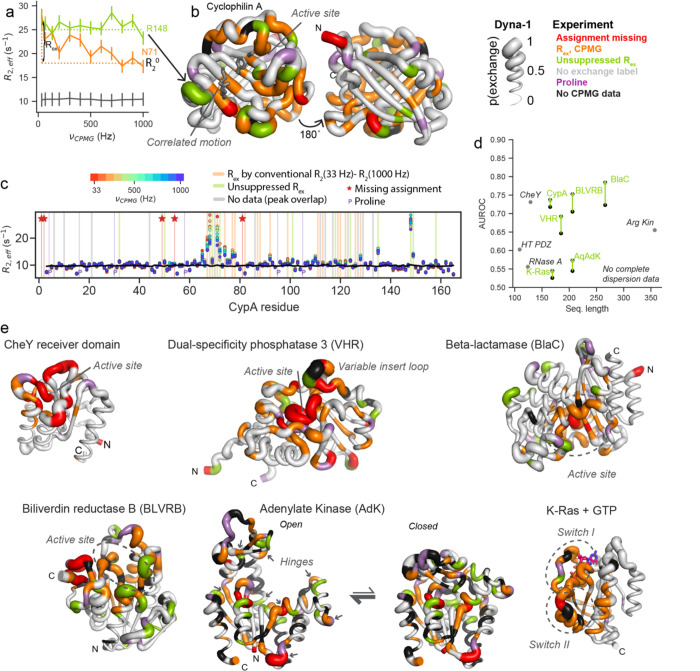
Dyna-1 even predicts exchange in residues that conventional CPMG data analysis misses. (a) Example ^15^N CPMG data for 3 residues from Cyclophilin A (CypA)^40^. Error bars represent background noise in spectrum. R_ex_ is typically estimated via difference in R_2,eff_ at lowest field strength and R_2_^0^ value to which R_2,eff_ plateaus. However, plotting R_2,eff_ for Arg148 (green) demonstrates different behavior: R_2,eff_ remains high over all B_1_ field strengths used, indicative of exchange on the microsecond regime that is too fast to be suppressed with the CPMG field strengths used. (b) AF2 structure of CypA with tube thickness by Dyna-1 p(exchange). Legend coloring according to the experimental data is shown to the right. Dyna-1 predicts high exchange for Arg148. (c) We devised a data-processing method using R_2_ predicted by HYDRONMR to systematically assign residues with R_ex_ for residues where R_2,eff_ get suppressed by CPMG pulse train (conventional R_ex_, in orange) or residues with elevated, unsuppressed R_ex_ (green). Spectra depicted with representative data for CypA was collected at 25°C. (d) Incorporating labels for unsuppressed R_ex_ increases reported AUROC for 5/6 proteins where complete dispersion data was available for reprocessing (shown by green arrows). Also shown is the AUROC for proteins where we could not find dispersion data for all residues, and instead curated residue labels based on descriptions in literature. (e) Representative experimental data and Dyna-1 predictions for a selection of proteins with dynamics characterized with ^15^N CPMG. For Adenylate Kinase, structure predictions of the open and closed states from AF2 were used as input into Dyna-1.

Due to these challenges in discerning different types of dynamics in single-field-strength R_1_/R_2_/hetNOE experiments, we lastly tested Dyna-1 on proteins with μs-ms exchange directly characterized by ^15^N CPMG relaxation dispersion (RelaxDB-CPMG dataset, Fig. 5). CPMG experiments allow for directly measuring contribution from conformational exchange to R_2,eff_^9,15,16^. In brief, data are collected with increasingly short gaps between spin-echo pulses, which increasingly suppress contributions from exchange between states with different chemical shifts (R_ex_) to R_2_^10,15,16^. In typical data processing (orange trace, Fig. 5a), R_2,eff_ plateaus at a value that can be understood to be R_2_ in the absence of chemical exchange (R_2,inf_). The difference between R_2,eff_ at the lowest B_1_ field strength and R_2,inf_ is the R_ex_ contribution to R_2_ that was suppressed. We note that because our RelaxDB-CPMG dataset is curated with ^15^N relaxation data, our evaluations on the experimental data do not consider cases where R_ex_ is only seen for ^1^H caused by chemical shift differences only in ^1^H and not ^15^N, therefore underestimating other possible chemical exchange. Hence, Dyna-1 performance might be better than reported.

We first evaluated Dyna-1 by creating labels for residues with exchange following this data processing. Fig. 5b depicts Cyclophilin A (CypA) sized by Dyna-1 prediction probabilities, where residues with statistically significant R_ex_ from ref. ^38^ are colored orange. Importantly, Dyna-1 predicted higher p(exchange) in the beta-sheet with a known concerted millisecond dynamics involving the sidechains of residues Ser99, Phe113, Met61, and Arg55^39^ (shown in grey in Fig. 5b). Note that the other beta-sheet on the back of CypA, is correctly predicted to have no exchange. We were curious about residue Arg148: Dyna-1 predicted high exchange probability, but Arg148 did not register as having significant R_ex_ as evaluated by the above method. When looking at the raw data, we found that Arg148 had high R_2,inf_ compared to other residues. We propose that Arg148 has exchange in a process that is too fast to be suppressed by the B_1_ fields used in the CPMG experiment for the given temperature. However, to confirm this requires a reasonable estimate for what R_2,inf_ should be if R_2,eff_ is never sufficiently suppressed by the CPMG field strength. We devised a new framework to estimate anisotropic effects from rigid tumbling to then identify unsuppressed R_ex_ (see Methods). Fig. 5c depicts CPMG data for CypA collected at 25°C and 600 MHz from a B_1_ field strength of 33 (red) to 1000 Hz (blue). Several residues have unsuppressed R_ex_ (in green) which conventional data processing would not have identified as having exchange.

We applied our new processing to 6 datasets where we had ^15^N CPMG dispersion data for all non-overlapped residues, including CypA^40^, beta-lactamase^21^, Adenylate Kinase from *Aquifex Aeolicus*^41^, Biliverdin reductase B^42^, K-Ras^43^, and Dual-specificity phosphatase 3^44^ (Extended Data Fig. 8). For all datasets, Dyna-1’s performance improved when considering residues with unsuppressed R_ex_ (Fig. 5d, structures in Fig. 5e). Dyna-1 also predicts unsuppressed R_ex_ in Biliverdin reductase B, a cellular redox regulator, where millisecond motions are implicated in efficient coenzyme engagement and catalysis^42^, and predicts concerted millisecond dynamics in the switch I and II regions of K-Ras that is well known to be crucial for its signaling activity^43^. We additionally analyzed 4 proteins where CPMG was performed but raw data was unavailable, and found that Dyna-1 had predictive power for residues other labs reported had exchange (Extended Data Fig. 9).

### Prospective experimental test of Dyna-1.

**Fig. 6:**
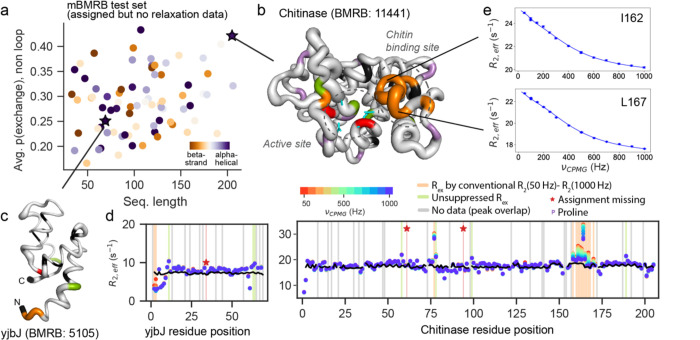
Dyna-1 has predictive power for enzymatic motions in prospective experimental test. a) Dyna-1 proteins in mBMRB test set vary in net p(exchange) predicted across sequence length and helix/strand content. We selected two proteins to experimentally test Dyna-1 predictions: (b) Chitinase 19 from *Bryum coronatum*, and (c) hypothetical protein yjbJ. Residue coloring in (b,c,d) is same as in Fig. 5. (d) ^15^N CPMG relaxation dispersion only showed dispersion curves for two N-terminal residues in yjbJ, whereas chitinase has extensive exchange in the active site and chitin binding site (see also supplemental dataset 1). (e) Representative ^15^N CPMG dispersion data for I162 and L167 in the chitin binding site region.

To further validate the predictive capabilities of Dyna-1, we conducted a prospective experimental test after public release of the model and initial review of the manuscript. We selected proteins from the “mBMRB test” set to measure their dynamics, as they are already assigned but many do not have published relaxation data. Plotting the mean p(exchange) from each protein in the mBMRB test set vs. sequence length demonstrates that the predicted mean p(exchange) from Dyna-1 is broad for any given sequence length or helix / strand content (Fig. 6a). For proteins at the top and bottom range of mean p(exchange), we discarded proteins as candidates if they were fully intrinsically disordered, did not have published expression/purification methods sections, and already had some relaxation data available. From the remaining proteins, we selected Chitinase 19 from *Bryum coronatum*^45^ (BMRB: 11441) with a high degree of p(exchange) (Fig. 6b), and hypothetical protein yjbJ^46^ (BMRB entry: 5105) with a low degree of p(exchange) (Fig. 6c). Performing ^15^N relaxation dispersion CPMG demonstrated that, resembling Dyna-1’s predictions, the chitinase did have exchange in regions that are crucial for biological activity—in its active site and the loops known to bind chitin (Fig. 6b,e)—while the yjbJ only had detectable exchange for residues 2 and 3 (Fig. 6c,d, supplemental dataset 1). Evaluated at a per-residue level, Dyna-1 obtained an AUROC of 0.62 for chitinase and an AUROC of 0.72 for yjbJ.

## Discussion

AF2 fundamentally altered the course of molecular biology and was made possible thanks to the clear task of protein structure prediction defined in the Critical Assessment for Structure Prediction (CASP) challenges and the large amount of standardized data deposited in the PDB. Though widely acknowledged that understanding protein dynamics is essential for understanding protein function, the experimental measurements of dynamics that have uncovered foundational principles of allostery, specificity, catalysis, and many more biological functions have resisted being collected into standardized datasets. Given the predictive power displayed in Dyna-1, it is essential that the field deposits experimental data on dynamics in macromolecules. One barrier to this in NMR has been the perceived diversity and complexity of experiments that measure dynamics. We have laid out in this work a clear task—classify residues with μs-ms exchange—and showed that we could train a model with predictive power across multiple experimental data types: missing assignments, R_ex_ from R_1_/R_2_/hetNOE, and multiple exchange indicators from CPMG.

Dyna-1’s predictive power learned from missing assignments was despite many issues with the current publicly-available data. The data that we used to obtain our labels, lists of atom assignments, are the end point of NMR data processing. The spectra themselves are not required to be deposited, and the lack of standardized requirements to deposit them is a major loss for science. Peak width is related to R_2_^47^, and hence if deposition of even one representative spectrum per assignment dataset had been required, the community could have had roughly the information content of RelaxDB (i.e., R_ex_ labels) for two orders of magnitude more proteins.

Further improvements to Dyna-1 doubtlessly exist for the task of classifying residue dynamics: for instance, ESM-3, the base of Dyna-1, was not trained with sidechain information, information that will likely improve performance. Additionally, Dyna-1 is trained on proteins under 400 residues in length, possibly limiting the scope in which Dyna-1 can predict the dynamics involved in larger proteins. Our comparison of the Dyna-1 model architecture with a training split of 30% sequence identity, 0.5 TM-score cutoff to a larger and more lenient training split of 80% sequence identity, 1.0 TM-score cutoff showed only a marginal increase in performance. This indicates that future model improvements are likely to require innovations in model architectures, rather than increasing data alone.

## Supplementary Material

Supplement 1

## Data Availability

The mBMRB training set, RelaxDB, and RelaxDB-CPMG datasets are publicly available for noncommercial use at https://github.com/WaymentSteeleLab/Dyna-1 and at https://huggingface.co/spaces/gelnesr/Dyna-1, doi: 10.57967/hf/9277. Raw data for CPMG relaxation dispersion for the two prospective proteins characterized in this study are deposited at 10.5281/zenodo.20834076.

## Code availability.

Code to run Dyna-1 is available at https://github.com/WaymentSteeleLab/Dyna-1 and https://huggingface.co/spaces/gelnesr/Dyna-1.
