# Supplementary material for: Learning millisecond protein dynamics from what is missing in NMR spectra
Source: bioRxiv. 2026 Jul 1:2025.03.19.642801. Preprint. [Version 3] doi: 10.1101/2025.03.19.642801 (PMC13345324; doi:10.1101/2025.03.19.642801)
Supplement: Supplement 1 [file NIHPP2025.03.19.642801v3-supplement-1.pdf]

bioRxiv preprint doi: https://doi.org/10.1101/2025.03.19.642801; this version posted July 1, 2026. The copyright holder for this preprint (which was not certified by peer review) is the author/funder, who has granted bioRxiv a license to display the preprint in perpetuity. It is made available under a CC-BY 4.0 International license.

Supporting Information

I. Thorough evaluation of reported NMR relaxation data and corresponding publications increases Dyna-1 performance

II. Methods

III. Extended Data Figures

bioRxiv preprint doi: https://doi.org/10.1101/2025.03.19.642801; this version posted July 1, 2026. The copyright holder for this preprint (which was not certified by peer review) is the author/funder, who has granted bioRxiv a license to display the preprint in perpetuity. It is made available under a CC-BY 4.0 International license.

Thorough evaluation of reported NMR relaxation data and corresponding publications increases Dyna-1 performance

While inspecting predictions for other proteins, we noticed that Dyna-1 predicted high p(exchange) for some residues with reported assignments but no relaxation data. This prompted us to revisit our initial curation of experimental data. For instance, in reduced Mercuric Transport protein (MerP)[35], Thr13 predicts high p(exchange), but has no relaxation data. Ref. [35] notes that the peak for Thr13 is very weak, which indicates exchange-broadening and we posit prohibited the authors from fitting R_1 and R_2. Though Ala15 was not labeled by our automated labeling scheme, it also has elevated exchange and a large error in R_2/R_1 (orange arrow, **Fig. 4c**). Adjusting the experimental label for both residues increases Dyna-1 AUROC from 0.98 to 1.

Another example is the Tudor domain of Survival of Motor Neuron protein (SMNTD)[36], some of the highest p(exchange) from Dyna-1 were in two residues that were assigned yet had no relaxation data reported: Glu104 and Tyr130 (depicted in black, **Fig. 4c**). The amide hydrogen of Glu104 was assigned at 4.9 ppm, which is a highly unlikely value for an amide hydrogen. We posit that for this small domain and given the well-resolved spectra that the authors report, this residue is more likely exchange-broadened with an incorrect assignment reported. For Tyr130, no R_1 value was reported, yet the authors categorized it as fitting to “model 4” from the model-free formalism[36], which is a model containing R_{ex} and requires an R_1 value. If we consider both residues as having evidence of exchange, Dyna-1’s AUROC increases from 0.67 to 0.75.

In the NtrC receiver domain, Dyna-1 achieved an AUROC of 0.57. We revisited the 10 residues that were assigned yet had no relaxation data. We found that 2/10 had been erroneously assigned in chemical shift depositions corresponding to ref. [37] despite being exchange-broadened. 5/10 had very small peaks, indicative of exchange-broadening, and the remaining 3/10 were unanalyzable due to peak overlap. Taking these into account increases Dyna-1’s AUROC to 0.60. Dyna-1 missed predicting exchange

bioRxiv preprint doi: https://doi.org/10.1101/2025.03.19.642801; this version posted July 1, 2026. The copyright holder for this preprint (which was not certified by peer review) is the author/funder, who has granted bioRxiv a license to display the preprint in perpetuity. It is made available under a CC-BY 4.0 International license.

in Tyr101 posited to be from ring-flipping. This is exemplary of what we found more broadly across all of RelaxDB, that Dyna-1 is weakest at predicting exchange in beta-sheets (**Fig. 4d**).

Another example is Tyrosine-phosphatase A from *M. tuberculosis* (MptpA)[41]. Dyna-1 has high p(exchange) in loops containing residues with differing orientations between the determined apo and holo structures (apo: 2LUO, holo: 1U2P) and which are postulated to be involved in regulating enzyme specificity[41]. Some of Dyna-1's high p(exchange) is in the P-loop of MptpA, which our curation indicated had been assigned (BMRB:18533) yet had no relaxation data (BMRB: 26513). However, the authors noted, "Due to exchange broadening the P-loop, residues Cys^{11}–Gly^{13} and Ile^{15}–Ser^{18} were not detectable"[41]. If we consider these labels, the AUROC for MptpA increases from 0.55 to 0.63.

bioRxiv preprint doi: https://doi.org/10.1101/2025.03.19.642801; this version posted July 1, 2026. The copyright holder for this preprint (which was not certified by peer review) is the author/funder, who has granted bioRxiv a license to display the preprint in perpetuity. It is made available under a CC-BY 4.0 International license.

Methods

Curating the "RelaxDB" dataset.

Data collected at one magnetic field strength results in three values per residue: a R_1, R_2, and hetNOE value. R_{ex} is the increase in R_2, the backbone amide's transverse relaxation (see Methods), caused by exchange between states with different chemical shifts on the µs-ms timescale. A common practice to estimate the presence of additional R_{ex} contribution to R_2 is to look for high outliers in R_2/R_1 per residue. R_1 does not contain R_{ex} and decreases if exchange is present at slower timescales. However, anisotropic tumbling can also alter the R_2/R_1 ratio. The model-free formalism[19-21] was developed to estimate R_{ex} from these data; however, we were unable to robustly apply it at this scale of datasets since relaxation data from multiple field strengths, important for accurate estimations, were often not available. Therefore, we developed the following processing method to label residues with R_{ex}. We generated AF2 models for the 163 proteins and calculated their R_2 and R_1 values accounting for anisotropic rigid-tumbling only with HYDRONMR[22,23], fitting one constant per protein that represents the ratio between the experimental and the HYDRONMR overall tumbling time. We then designated a residue as having R_{ex} present if the difference between experimental R_2 and rigid-tumbling R_2 (dR_2) was greater than the combined modelling and experimental uncertainty for that residue (see Methods). We also labeled residues as having ps-ns motion if their hetNOE was less than 0.65 [24-26].

Theoretical basis for relaxation measurements. The following is condensed from ref. [1]. The longitudinal (R_1) and transverse (R_2) relaxation rates of a backbone nitrogen atom attached to a single proton may be written in a simplified form as

$$R_1 = \frac{A}{10} J(\omega_N)\,,$$

$$R_2 = \frac{A}{15}\left[J(0) + \frac{3}{4} J(\omega_N)\right] + R_{ex},$$

bioRxiv preprint doi: https://doi.org/10.1101/2025.03.19.642801; this version posted July 1, 2026. The copyright holder for this preprint (which was not certified by peer review) is the author/funder, who has granted bioRxiv a license to display the preprint in perpetuity. It is made available under a CC-BY 4.0 International license.

where $J(\omega) = S^2\tau_C/(1+\omega^2{\tau_C}^2)$. $J(\omega)$ is the spectral density function describing the intensity of exchange occurring at frequency ω. These expressions do not include terms on the order of $J(\omega_H)$, which are small on the timescale of protein tumbling ($\omega_N\tau_C \gg 1$). In the above, $A = 3d^2 + 4c^2$, where d=$\frac{\mu_0\, h\, \gamma_H\, \gamma_N}{r_{NH} 8\pi^2}$ and $c = \frac{\gamma_N B_0 \Delta\sigma}{\sqrt{3}}$. μ_0 is the permeability of free space, *h* is Planck's constant, γ_X is the gyromagnetic ratio of atom X (nitrogen N or hydrogen H), r_{NH} is the bond length of the N-H bond, B_0 is the static magnetic field strength, and $\Delta\sigma$ is the chemical shift anisotropy of nitrogen.

Note that the ratio R_2/R_1 for residues that do not demonstrate intramolecular dynamics can be used to estimate the global tumbling time τ_C via the following relationship:

$$\frac{R_2}{R_1} = \frac{2}{3}\frac{J(0)}{J(\omega_N)} + \frac{1}{2} = \frac{2}{3}{\omega_N}^2{\tau_C}^2 + \frac{7}{6}.$$

RelaxDB curation. We curated 163 R_1/R_2/NOE datasets in total. Roughly half of these were deposited in the BMRB, though many required further curation such as converting from incorrectly deposited units. The other half came from supplemental tables of published papers, plot digitization from figures in publications, and private correspondences, including a benchmark of 20 proteins compiled by Goodman et al. in 2000[18].

BMRB entries.

To get entries from the BMRB that contained R_1, R_2 and hetNOE data, we used https://bmrb.io/software/query/ to download a list of all entries with one entity of polymer type polypeptide(L) that contained R_2 relaxation data (referred to as T_2 in the user interface). This resulted in 344 entries when queried in January 2023. We manually went through the list and identified entries that were monomers, had no cofactors present, were not in micelles/bicelles/nanodisks, were not in

bioRxiv preprint doi: https://doi.org/10.1101/2025.03.19.642801; this version posted July 1, 2026. The copyright holder for this preprint (which was not certified by peer review) is the author/funder, who has granted bioRxiv a license to display the preprint in perpetuity. It is made available under a CC-BY 4.0 International license.

denaturing conditions, and had at least one set of R_1/R_2/hetNOE data all at the same field strength. This resulted in 91 entries. We included data collected at multiple field strengths when available. Several of the BMRB entries had incorrect units, which we fixed by visually inspecting data and comparing to figures of plotted data in the corresponding publications. For three entries, we realized that they contained two or more domains, but that these two or more domains could be modelled as tumbling independently. We therefore separated these into two separate entries.

Data from literature.

To find data from literature, we searched in PubMed and Google Scholar for keywords "NMR", "backbone", and "relaxation". Upon finding a dataset of an apo, monomeric, stably folded protein and if the data was only available in figure form, we used PlotDigitizer (https://plotdigitizer.com/) to compile values for R_1/R_2/hetNOE. We did not include datasets if the figures were too low-resolution to allow for accurately using PlotDigitizer. We only used datasets where we could identify a corresponding set of assignments either cited in the same paper or clearly referencing another set of assignments.

Fitting data / assigning labels.

Generating MSAs.

We generated MSAs using the `run_mmseqs2`command in ColabFold with `filter=True`. We used these MSAs to predict AF2 models. Prior to calculating conservation metrics (below), we removed sequences with more than 10% gaps and used MMseqs easy-cluster to further filter with options `-c 0.9 --cov-mode 1 --min-seq-id 0.9`.

Generating structure models.

bioRxiv preprint doi: https://doi.org/10.1101/2025.03.19.642801; this version posted July 1, 2026. The copyright holder for this preprint (which was not certified by peer review) is the author/funder, who has granted bioRxiv a license to display the preprint in perpetuity. It is made available under a CC-BY 4.0 International license.

We used AF2 as implemented in ColabDesign (https://github.com/sokrypton/ColabDesign.git@gamma) to generate the models used for HYDRONMR calculations. We ran AF2 for 3 recycles, seed=0. We used model 1 for each protein. For the Adenylate Kinase datasets (both from *E. coli* and *A. Aquifex*), AF2 predicts the closed state though it is known to occupy the open state predominantly in solution. Therefore, we templated AF2 using structure model PDB ID 4AKE of the open state.

We used these structure models as inputs into HYDRONMR[2,3] with default settings to estimate R2/R1 from rigid tumbling, denoted as $\widehat{R_2^0/R_1^0}$. We trimmed unstructured termini of the 161 proteins by removing residues from the N or C termini until the pLDDT of the next residue was over 70, or over 80 for CBFB and PCF11. Residues corresponding to unstructured termini are not considered during evaluation.

Fitting R_2/R_1 to rigid tumbling estimates.

We needed to account for a constant offset in the overall tumbling time between each experimental dataset and the corresponding prediction. To do so, we fit $\widehat{R_2^0/R_1^0}$ to the experimental data R_2/R_1 as follows: for each dataset, we wish to minimize the squared difference between R_2/R_1 and $C\ \widehat{R_2^0/R_1^0}$ where C is a scalar to be fit, i.e.:

$$\min_C \frac{1}{n} \sum_{i=1}^{N} \left[\frac{R_2}{R_1}(i) - C\,\widehat{\frac{R_2^0}{R_1^0}}(i)\right]^2 .$$

This has a closed-form solution for C:

$$C = \frac{\sum_i \frac{R_2}{R_1}(i) \times \widehat{\frac{R_2^0}{R_1^0}}(i)}{\sum_i \widehat{\frac{R_2^0}{R_1^0}}(i)^2}.$$

bioRxiv preprint doi: https://doi.org/10.1101/2025.03.19.642801; this version posted July 1, 2026. The copyright holder for this preprint (which was not certified by peer review) is the author/funder, who has granted bioRxiv a license to display the preprint in perpetuity. It is made available under a CC-BY 4.0 International license.

To avoid potential influence of outliers, we first calculate C excluding residues with hetNOE <0.65 as used in refs. [4-6] to designate residues with ps-ns motion. We use this C to calculate residuals (i.e., $\frac{R_2}{R_1}(i) - C\frac{\widehat{R_2^0}}{R_1^0}(i)$), then calculated C again excluding residues with a residual greater than the interquartile range.

Residues exhibiting R_2/R_1 greater than that predicted by rigid tumbling, i.e. R_{ex}, were identified as those with dR_2/R_1 > experimental error + fitting error. Fitting error for each protein was estimated as the interquartile range of the residuals, as this is more robust to outliers than a standard deviation. Experimental error for each residue was taken as the maximum of 5% of the reported R_2/R_1 value or the reported error in R_2/R_1, whichever value was larger. We also assigned a label for residues with ps-ns motion as residues with hetNOE < 0.65 as used in ref. [4-6] to designate residues with ps-ns motion.

All final datasets and corresponding HYDRONMR fits are depicted in **Extended Data Fig. 1**, as well as the normalized RMSE over all R_2/R_1 values for residues that were not assigned to have low hetNOE or elevated R_2/R_1. RMSE alone would be larger for larger proteins, so we report RMSE that is normalized to the mean R_2/R_1 value for each protein. Distributions of norm. RMSE for the final RelaxDB evaluation set, datasets which were initially curated but then excluded, and datasets of proteins removed for containing phosphate buffer and phosphate-related biological function are depicted in **Extended Data Fig. 2a**. In the final dataset, 65/111 datasets had a norm. RMSE of less than 0.1, and 107/111 had a norm. RMSE of less than 0.2 (**Extended Data Fig. 2b**). A norm. RMSE of 0.1 can be interpreted as the experimental data for residues that did not have dynamics assigned were on average within 10% of the predicted HYDRONMR value for R_2/R_1.

Filtering datasets resistant to fitting protocol.

After curating 163 datasets initially, we realized in the process of finding a standardized way to fit all of them that some would not be well fit for a variety of reasons. For instance, datasets ISDHN3,

bioRxiv preprint doi: https://doi.org/10.1101/2025.03.19.642801; this version posted July 1, 2026. The copyright holder for this preprint (which was not certified by peer review) is the author/funder, who has granted bioRxiv a license to display the preprint in perpetuity. It is made available under a CC-BY 4.0 International license.

MECP2, CBP6C, 5330, and MH35LIF were missing many R_2 and R_1 values, datasets 19356, CBP6N, and CBP6C all had hetNOEs < 0.65, and datasets 18773, 6758, and 5762 had relatively high Norm. RMSE values (see **Extended Data Fig. 2a** for comparison of all). We show the raw data for all the excluded datasets in **Extended Data Fig. 1**.

Per-residue analysis.

For the RelaxDB proteins and the analysis shown in **Fig. 1e**, we calculated conservation scores following the method described in ref. [7]. To determine buried/exposed residues, we calculated solvent accessible surface area (SASA) using the Shrake-Rupley method[8] implemented in MDtraj[9] and normalized values by the maximum value per secondary structure type (loop, helix, sheet) over the whole dataset, following the practice in ref. [10]. We assigned residues with a normalized SASA ≤ 0.2 as core and > 0.2 as surface residues, following ref. [10].

The "mBMRB" dataset

Curating data.

We downloaded a list of all entries consisting of polymer type polypeptide(L) from the BMRB[11] using https://bmrb.io/software/query/. We processed this to retrieve all entities within each entry which were type polypeptide(L). On May 31, 2023, this resulted in 12102 entities. BMRB entries were downloaded using pynmrstar v3.3.1.

We removed entities with isotope labels corresponding to deuterated samples, as a deuterated sample that has not completely back-exchanged with H_2O would lead to missing assignments. We also removed methyl-labeled samples or samples otherwise partially labeled based on keywords in the isotope metadata. Additionally, we removed any sequences shorter than 30 residues. After this filtering, we had 10123 entities.

bioRxiv preprint doi: https://doi.org/10.1101/2025.03.19.642801; this version posted July 1, 2026. The copyright holder for this preprint (which was not certified by peer review) is the author/funder, who has granted bioRxiv a license to display the preprint in perpetuity. It is made available under a CC-BY 4.0 International license.

We removed any entities with “X” in the sequence (242 removed). Some sequences deposited included multiple copies if the entity consisted of a multimer. We detected and edited these sequences to contain only one copy of the monomeric subunit to match the deposited data. We realized that 1834 sequences contained His-tags, many of which were unassigned. We therefore masked the N- or C-termini of sequences if 6 or more contiguous histidines were present, including everything before the His tag if it was an N-terminal His tag, or everything after if it was a C-terminal His tag. We further filtered the remaining entities to include only entities with 10 or more assignments, to remove entries that included only a few backbone chemical shifts (removed 273).

For each residue, we assigned labels corresponding to whether the ^{15}N backbone chemical shift is missing or not based on if that residue had a chemical shift entry corresponding to an “Atom_ID” of N.

We further removed entities that had 15 or more contiguous missing assignment labels between any assigned residues. This was to filter entities where we noticed that entire domains were unassigned. We selected the cutoff of 15 contiguous residues because the most residues missing in a row in the RelaxDB dataset was 14. This removed 227 entities.

Of the remaining 9,381 entries, 5% did not have isotope information present. However, we elected to keep these, as we saw that they had on average fewer missing assignments and shorter sequences than the 95% of other remaining sequences which had 1H labeling (**Extended Data Figure 3a,b**). Roughly 60% of these are older entries (entry_ID < 10000).

Extended Data Fig. 3d contains average fraction missing across the mBMRB divided by pH, temperature, and sequence length. From this we observe that there are certain well-known factors that play a role in

bioRxiv preprint doi: https://doi.org/10.1101/2025.03.19.642801; this version posted July 1, 2026. The copyright holder for this preprint (which was not certified by peer review) is the author/funder, who has granted bioRxiv a license to display the preprint in perpetuity. It is made available under a CC-BY 4.0 International license.

more missing assignments—for instance, pH greater than 8, or sequence length greater than 250—but these entries also represent small fractions of the data in total. Understanding how to more systematically account for such systematic outliers will be the topic of future research.

Structure models for each protein in the mBMRB dataset were generated using ESMFold v1[12].

Comparing to structure models in PDB

We used MMseqs[13] to align all the mBMRB sequences (9,381) , filtered to 80% sequence identity, against all protein chains in the PDB (curated based on PDB data in NMRbox, October 2024). We kept all hits with a bitscore greater than 50 and sequence identity greater than 90%. We kept one chain per protein sequence per model. We found that 95% of the mBMRB had one or more similar PDB structures.

RelaxDB-CPMG curation

Complete dispersion data available

AdK: $R_{2,eff}$ vs ν_{CPMG} for apo AdK from *Aquifex aeolicus* at 10℃ collected at 800 MHz, presented in ref. [14], as well as a corresponding peak list, was found in the Kern lab data archive, with thanks to K. Henzler-Wildman.

BlaC: $R_{2,eff}$ vs ν_{CPMG} of apo BlaC collected at 600 MHz and 850 MHz, presented in ref. [15], was kindly provided by M. Ubbink. A peak list to identify missing assignments was taken from BMRB entry 27888 (same as in RelaxDB).

BLVRB: $R_{2,eff}$ vs ν_{CPMG} of apo BLVRB, presented in ref. [16], was kindly provided by E. Eisenmesser. A peak list to identify missing assignments was taken from BMRB entry 27462.

bioRxiv preprint doi: https://doi.org/10.1101/2025.03.19.642801; this version posted July 1, 2026. The copyright holder for this preprint (which was not certified by peer review) is the author/funder, who has granted bioRxiv a license to display the preprint in perpetuity. It is made available under a CC-BY 4.0 International license.

CypA: $R_{2,eff}$ vs ν_{CPMG} of apo CypA, collected at 10℃ and 25℃ at 600 MHz in ref. [17] , as well as peak lists to identify missing assignments at both temperatures, were found in the Kern lab data archive. Because different processes are more prominent at 10℃ or 25℃, the final labels created represent whether a residue experienced exchange at either temperature.

VHR: $R_{2,eff}$ vs ν_{CPMG} was taken from 800 MHz data provided in Table 7.10.3 of ref. [18]. Missing assignments were taken from assignments in Table 7.10. Note that these differ from assignments by the authors in BMRB 27950; the assignments in the dissertation table are more complete.

K-Ras: $R_{2,eff}$ vs ν_{CPMG} of K-Ras with GTP bound, presented in ref. [19], had been publicly deposited at https://doi.org/10.5061/dryad.j6q573nm0.

Complete dispersion data unavailable

CheY: Residues with missing assignments were taken as the black residues in Fig. 2 from ref. [20]. Residues were labeled as having exchange when CPMG could individually be fit (all residues in Table 2 in ref. [20]). This is CheY in the presence of 1 mM EDTA to remove any Mg^{2+}, which alters the dynamics.

HtrA2 PDZ domain: Values for R_{ex} were taken from source data corresponding to Fig. 2a in ref. [21]. Assignments were taken from BMRB:51320.

RNase: Residues were labeled as having exchange when R_{ex} >3 Hz at 20℃ in either 500MHz or 600 MHz data. This recreates Fig. 4B in ref. [22]. The $R_{2,eff}$ vs ν_{CPMG} data for these residues was kindly provided by P. Loria, but the dispersion data for all residues was not available.

bioRxiv preprint doi: https://doi.org/10.1101/2025.03.19.642801; this version posted July 1, 2026. The copyright holder for this preprint (which was not certified by peer review) is the author/funder, who has granted bioRxiv a license to display the preprint in perpetuity. It is made available under a CC-BY 4.0 International license.

Arginine kinase: Labels for residues with exchange were taken from Table 1 in ref. [23], which summarized all residues for which CPMG data could be fit individually. Table S1 in ref. [23] indicated if residues were missing due to exchange-broadening or overlap, and we used those labels.

Identifying unsuppressed R_{ex} in CPMG experiments

We first compared model predictions to labels for exchange where we assigned labels using a conventional assignment from the literature: any residue where R_{ex} is significantly apparent, calculated as $R_{2,eff}$ (first field strength) - $R_{2,eff}$ (last field strength) is greater than background noise.

However, we noticed that in some datasets there were residues with noticeably high $R_{2,eff}$'s across all field strengths but $R_{2,eff}$(first) - $R_{2,eff}$(last) was not substantial enough to be greater than 2-3 Hz. These correspond to residues that have exchange at the microsecond timescale: at the fast end of the micro-millisecond regime that CPMG can detect.

However, assigning these residues with confidence requires determining what residues have elevated $R_{2,eff}$ when there is no suppression, the equivalent of measuring R_{ex} as is conventionally done. Given our success using HYDRONMR to quantitatively fit R_2/ R_1 ratios in RelaxDB, we used HYDRONMR to calculate R_2values and fit a scalar to find the closest fit between $R_{2,inf}$ and $R_{2,rigid}$.

The $R_{2,eff}$ data for all residues, corresponding $R_{2,rigid}$ values, and labels are in **Extended Data Fig. 8**.

Data splitting

bioRxiv preprint doi: https://doi.org/10.1101/2025.03.19.642801; this version posted July 1, 2026. The copyright holder for this preprint (which was not certified by peer review) is the author/funder, who has granted bioRxiv a license to display the preprint in perpetuity. It is made available under a CC-BY 4.0 International license.

We clustered sequences using MMseqs[13] `easy-cluster` with arguments `--cluster-mode 1 --min-seq-id 0.8 -k 5`. We used the resulting clusters to remove all sequences similar to those in RelaxDB or RelaxDB-CPMG. From the remaining sequences, we randomly separated out 500 clusters for the validation set and 100 clusters for the test set.

For training with a 50% or 30% sequence identity cutoff, we used –min-seq-id 0.5 [or 0.3] to cluster but kept the same proteins in the validation and test set. To create training splits with structure homologues removed at TM-score = 0.5 or 0.7, we calculated all pairwise TM-scores using TMalign[24] between the mBMRB-Train set and mBMRB-Val, mBMRB-Test, RelaxDB, and RelaxDB-CPMG sets. We used TM-score normalized by the average length of the query and target protein, as recommended in ref. [24].

The model sweep depicted in **Fig. 3c** was performed with a different dataset split that considered all 163 sequences initially curated for RelaxDB and used a split of 80% sequence identity, 1.0 TM-score cutoff. The splits used for the model sweep in **Fig. 3c** can be found in the `oct2024` data repository. Subsequent model training was reperformed using a data split considering only the later filtered 133 sequences in RelaxDB and used the most stringent training data split (30% sequence identity, 0.5 TM-score cutoff). These subsequent models are depicted in **Figs. 3d** and onwards.

Baseline training

Our baseline training aimed to test simpler representations of protein sequences and/or structure. We use the mBMRB data to train these methods. The results of these baseline models on the mBMRB-Validation, mBMRB-Test, and RelaxDB datasets are depicted in **Extended Data Fig. 4a**. None outperform the models based on pre-trained models.

bioRxiv preprint doi: https://doi.org/10.1101/2025.03.19.642801; this version posted July 1, 2026. The copyright holder for this preprint (which was not certified by peer review) is the author/funder, who has granted bioRxiv a license to display the preprint in perpetuity. It is made available under a CC-BY 4.0 International license.

Naïve baselines: The simple, naïve baseline uses amino acid frequencies in the mBMRB training data as weighting of a random classifier.

- The simplest is the AA baseline, which computes the frequency of each amino acid being missing from the mBMRB training data. We then predict for our validation and test set whether a residue is missing at random, weighted by that relative frequency.
- We evaluate similar random classifier using frequency of secondary structure (loop, sheet, helix) as calculated by DSSP, and a combination termed the "AA & DSSP" baseline using the frequency of each amino acid type given its secondary structures (loop, sheet, helix).

Simple classifiers: We use a similar Transformer classifier as that described for the final Dyna-1 architecture trained on simpler representations of the input mBMRB training data. Each representation is described.

- "OHE AA": we input each protein as a one-hot encoded sequence, where each residue is represented by a unique combination of 0s and 1s, into the transformer classifier.
- "SASA" we evaluate solvent-accessible surface area using the Shrake-Rupley method[8] implemented in MDTraj[9]. These values are encoded by a simple one-dimensional MLP of hidden size 128 and then input into the transformer classifier.
- The "SASA & DSSP", "SASA & AA", and "SASA & AA & DSSP" concatenate the outputs of the MLP with a one-hot encoding of the secondary structure, sequence, and secondary structure and sequence, respectively. This concatenated embedding is subsequently used as input into a transformer and trained accordingly.

Other representations:

- Normal mode analysis (NMA) is a computational technique that has long been of interest for modelling dynamic processes in proteins[25]. To evaluate NMA, we use ProDy[26] to compute the top 20 normal modes of the models generated using ESMFold (for the mBMRB data) or AF2 (for the

bioRxiv preprint doi: https://doi.org/10.1101/2025.03.19.642801; this version posted July 1, 2026. The copyright holder for this preprint (which was not certified by peer review) is the author/funder, who has granted bioRxiv a license to display the preprint in perpetuity. It is made available under a CC-BY 4.0 International license.

RelaxDB data) using the Anisotropic Network Model (ANM) and Gaussian Network Model (GNM) and build the Hessian matrix and Kirchhoff matrix, respectively. We then calculate the mean square fluctuation and evaluate the AUROC and AUPRC using these values. No model is trained.

- To use sequence conservation as a baseline, we computed sequence conservation scores and coverage index per protein as described in ref. [7] and evaluated the AUROC and AUPRC using these values as logits. No model is trained.

Deep learning training

We trained Dyna-1 on the mBMRB-Train dataset, sampling using a sequence-cluster-based approach. One protein per sequence cluster is chosen randomly during each epoch. We used the validation set to compute the validation loss and validation metrics. The final model weights for each training run for all models are chosen using max AUROC on the validation set. We excluded unassigned termini and prolines (which have no backbone amide and therefore are always missing) from model evaluations.

We used the ESM2-t30-150M model for ESM-2 embeddings, which has 31 layers and 150M parameters. Initial attempts using ESM2-t6-8M and ESM2-t12-35M indicated that smaller models did not perform as well, and ESM2-t33-650M performed marginally similar. We used the outputs of AF2 model 1 to construct the AF-pair representation. The weights from ESM and AF2 were fixed (frozen) during training.

Models were trained with the AdamW optimizer[27] with a learning rate of 1e-6. The AF2-pair model was trained with batch size of 4 with an accumulation step every 4 epochs, while the ESM-2 and ESM-3 based models were trained with a batch size of 16 and accumulation each epoch. Training is completed within 500 epochs, and we use early stopping when validation loss begins to overfit. We choose our best performing model by comparing normalized AUPRC on the validation set, as we saw this had slightly

bioRxiv preprint doi: https://doi.org/10.1101/2025.03.19.642801; this version posted July 1, 2026. The copyright holder for this preprint (which was not certified by peer review) is the author/funder, who has granted bioRxiv a license to display the preprint in perpetuity. It is made available under a CC-BY 4.0 International license.

more discrimination than AUROC. Normalized AUPRC is calculated by subtracting the baseline AUPRC from the AUPRC for each protein.

The transformer architecture used for the ESM-2 embeddings has 8 heads and 12 layers. The transformer for the ESM-3 embeddings has 6 heads and 12 layers, and the transformer for AF2-pair representation has 4 heads and 4 layers. We note that increase in number of heads or number of layers had negligible improvements for ESM-based models, and we were limited by GPU memory for AF2-pair. The hidden dimension for the ESM-based models is equivalent to the hidden size of the embedding, while for the AF2-pair model is 128. We apply a 0.1 dropout rate. All models were trained on a single NVIDIA A40 GPU, which takes between 12 hours and 2 days to complete training.

Adjusting logits to account for class imbalance.

When converting from logits to probabilities, we adjusted the raw logit outputs predicted by the model to account for the significant class imbalance in the problem following the practice in ref. [28] (cf. **Supporting Information Fig. 1**). This practice seeks probabilities that minimizes the "balanced error", the prediction error averaged over all classes. In brief, for data *x* labelled with classes *y*, the native posterior probability *P(y | x)* is proportional to the prior probability of class y, $\pi(y)$:

$$P(y|x) \propto \pi(y)P(x|y).$$

In seeking to minimize the balanced error, the balanced class-probability function instead implicitly becomes

$$P^{bal}(y|x) \propto P(x|y),$$

where *L* is the number of classes (L=2 in our binary classification context).

bioRxiv preprint doi: https://doi.org/10.1101/2025.03.19.642801; this version posted July 1, 2026. The copyright holder for this preprint (which was not certified by peer review) is the author/funder, who has granted bioRxiv a license to display the preprint in perpetuity. It is made available under a CC-BY 4.0 International license.

Menon et al. describe how $P^{bal}(y|x)$ is a Bayes-optimal scorer for minimizing the balanced error. For a Bayes-optimal scorer, the accuracy of any given class is not proportional to its degree of representation in the training data, which is desirable in our context.

Consider our model to be a scoring function $\hat{s}$, with learned class probabilities $\hat{P}(y|x) \propto e^{\hat{s}(x)}$. Via the definition of P^{bal}, we can approximate the Bayes-optimal scorer using our given scorer by using an adjusted scorer s':

$$P^{bal}(y|x) \approx e^{s'(x)} = \frac{\hat{P}(y|x)}{\pi(y)} = \frac{e^{\hat{s}(x)}}{\pi(y)}.$$

Accordingly, the adjusted logits may be written as $s'(x) = s'(x) - \log \pi(y)$.

In the context of our binary classification problem, let π^m be the fraction missing in the training data set and let *l(x)* be the raw logit output given input *x*. Dyna-1 is structured to just predict one logit, the logit of the "missing" class, hence the logit predicted for the "not missing" class is always zero. We calculate our probability P(missing) as

$$P(missing|x) = \frac{p(missing|x)}{p(present|x)+p(missing|x)} = \frac{e^{l(x)-\log \pi^m}}{e^{-\log(1-\pi^m)}+e^{l(x)-\log \pi^m}} = \frac{e^{l(x)}}{\frac{\pi^m}{1-\pi^m}+e^{l(x)}}.$$

This can be rewritten in terms of the sigmoid function $\sigma(x) = (1 + e^{-x})^{-1}$, which is typically used to convert logits to probabilities:

$$P(missing|x) = \sigma(x - \log \frac{\pi_m}{1-\pi_m}).$$

bioRxiv preprint doi: https://doi.org/10.1101/2025.03.19.642801; this version posted July 1, 2026. The copyright holder for this preprint (which was not certified by peer review) is the author/funder, who has granted bioRxiv a license to display the preprint in perpetuity. It is made available under a CC-BY 4.0 International license.

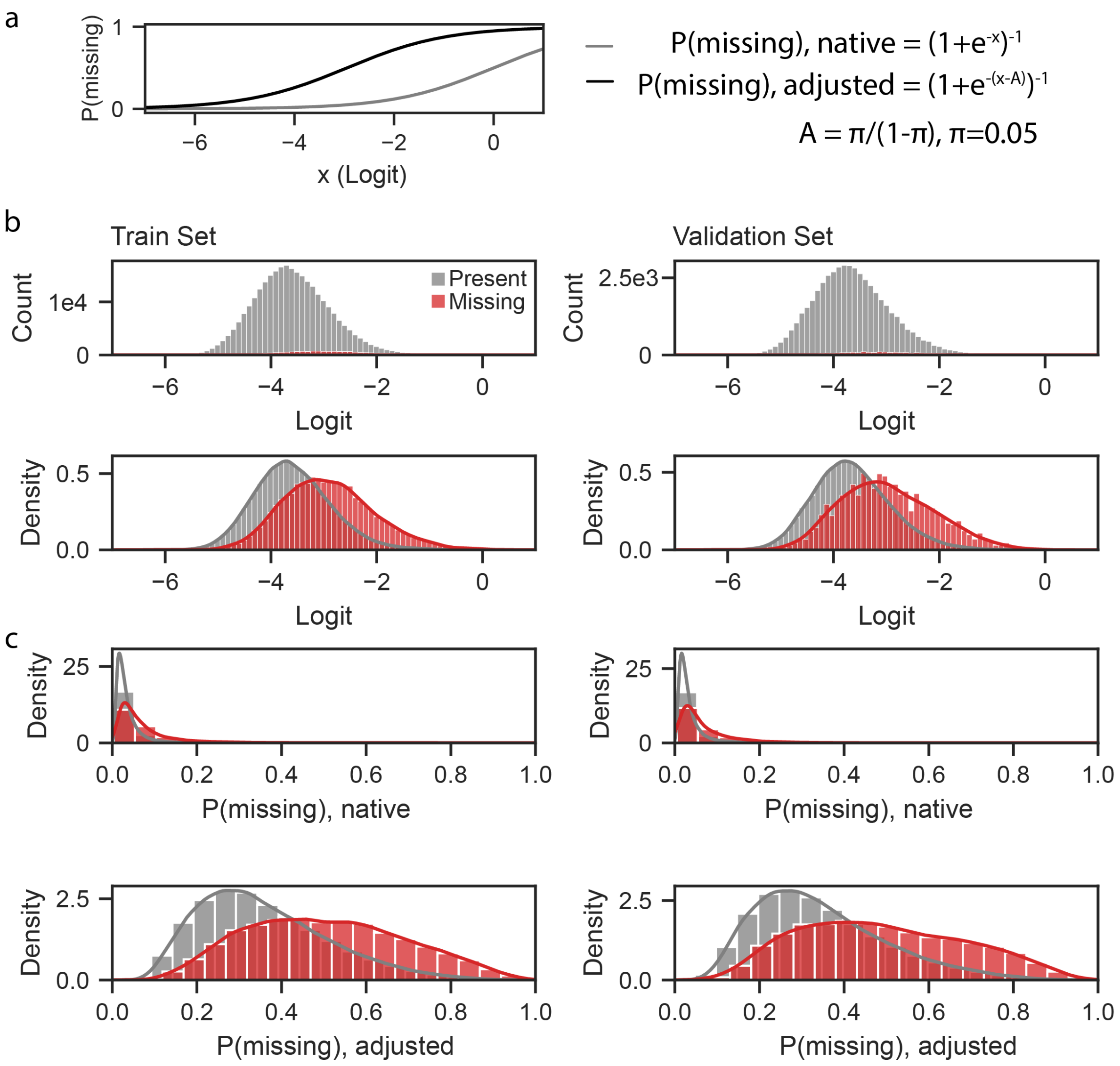

Supporting Information Figure 1: Adjusting native logits from Dyna-1 to account for class imbalance. (a) We adjusted probabilities calculated from logits to maximize the "balanced accuracy", i.e. accuracy averaged across both classes, rather than accuracy averaged over all datapoints (see Methods). We set our prior for the missing class based on the training data, π, as 5%. Comparing the adjusted curve (black) to the probability densities in (b) demonstrates how this intuitively matches where a discriminator between the two classes should be (at x ~ -3). (b) Histograms and probability densities of logits predicted for residues labeled as "present" and "missing" over train and validation sets. (c) Densities of resulting p(missing) if calculated using native logits or adjusted logits.

Uncertainty estimation.

bioRxiv preprint doi: https://doi.org/10.1101/2025.03.19.642801; this version posted July 1, 2026. The copyright holder for this preprint (which was not certified by peer review) is the author/funder, who has granted bioRxiv a license to display the preprint in perpetuity. It is made available under a CC-BY 4.0 International license.

We estimated uncertainty for comparing models in the RelaxDB dataset (**Fig. 4a, Extended Data Fig. 4**) using a 95% confidence interval calculated in Seaborn v0.11.2 [29].

We estimated uncertainty in binned residue analyses (**Fig. 4e,f**) using bootstrapping. We resampled residue subsets with replacement to obtain 100 bootstrap iterations that contained at least one of each class (required for calculating AUROC).

All depicted p-values (**Fig. 1e, Fig. 2h**) are for a 2-sided hypothesis test whose null hypothesis is that two sets of data are uncorrelated, performed using statannotations v0.2.3 (https://pypi.org/project/statannotations/).

We curated uncertainties for the RelaxDB datasets in the following way: if authors reported R_1/R_2/hetNOE uncertainties, we used those. In the BMRB, we found a few with a deposited uncertainty of zero. We set these to be the smallest nonzero uncertainty value given for that data type in the dataset. If no uncertainties were given, we set the uncertainty for R_1 as 10% the measured value, and 5% for R_2 and hetNOEs. If the data was plot digitized and had error bars, we digitized the error bars as uncertainties.

Preparation of recombinant proteins

The hypothetical protein yjbJ (YjbJ_ecoli) (NCBI: WP_058905752.1) and wild-type *Bryum coronatum (*BcChi-A) constructs were ordered from GenScript (Supplementary Table 1). The codon-optimized YjbJ_ecoli sequence was cloned into a pET-15b expression vector with an N-terminal 6xHis tag followed by a TEV protease cleavage site. The wild-type BcChi-A (without any affinity tag) was cloned into a pET-

bioRxiv preprint doi: https://doi.org/10.1101/2025.03.19.642801; this version posted July 1, 2026. The copyright holder for this preprint (which was not certified by peer review) is the author/funder, who has granted bioRxiv a license to display the preprint in perpetuity. It is made available under a CC-BY 4.0 International license.

22b vector between the NdeI (5') and BamHI (3') restriction sites, replacing the pelB leader sequence of the vector.

The individual plasmids encoding each protein were transformed into *E. coli BL21(DE3)* cells (New England Biolabs). Uniformly ^{13}C, ^{15}N-labeled proteins were expressed in *E. coli* grown in 1L M9 minimal medium supplemented with 2.0 g/L U-^{13}C as the main carbon source and 1.0 g/L of $^{15}NH_4Cl$ (Cambridge Isotope Laboratories). Cells were grown to an OD_{600nm} of 0.6 before induction with 1 M isopropyl thiogalactoside (IPTG). Then grown overnight at 25°C for the BcChi-A construct or 15 °C for the YjbJ construct for ~18 h.

Both constructs were purified using a similar method as previously described [30,31]. Briefly, YjbJ cell pellets were resuspended in 20 mM Tris, pH 8, 500mM NaCl, 30mM Imidazole (Buffer A), DNAse 1 (Sigma-Aldrich), and lysozyme (Sigma-Aldrich). Lysate was sonicated on ice for 10 min (20 sec on, 30 sec off, output power of 40 W), followed by clarification via centrifugation at 19000 rpm for 45 min at 4°C. A 5 ml His-trap Ni^{2+} affinity column (Cytiva) was pre-equilibrated in buffer A. The column was washed with 10 column volumes of buffer A after loading the supernatant, followed by elution of the his-tagged YjbJ protein with buffer containing 500 mM imidazole. The His tag was cleaved off with TEV protease and removed by loading the cleavage reaction back onto the His-trap Ni^{2+} affinity column. YjbJ with no tag was collected in the flow-through with 5 column volumes of buffer A containing no Imidazole. The ^{13}C,^{15}N labeled YjbJ was further purified on a Superdex 75 26/600 pg column in 25 mM Na_2PO4, pH 6.5. BcChi-A construct was purified by resuspending the pellet in 20mM Tris-HCl, pH 7.5, and sonicated on ice. The lysate was spun down at 18500 rpm for 45 min, followed by dialysis of the supernatant against 10 mM sodium acetate, pH 4 and additional clarification by spinning down at 18500 rpm for 20 min. The supernatant was then dialyzed in 10 mM sodium acetate, pH 5, before loading on a 5 ml HiTrap Q HP

bioRxiv preprint doi: https://doi.org/10.1101/2025.03.19.642801; this version posted July 1, 2026. The copyright holder for this preprint (which was not certified by peer review) is the author/funder, who has granted bioRxiv a license to display the preprint in perpetuity. It is made available under a CC-BY 4.0 International license.

column (Cytiva) pre-equilibrated in the same dialysis buffer. The protein was eluted with a linear gradient of NaCl from 0 to 150 mM NaCl in the same buffer. The ^{13}C, ^{15}N labeled BcChi-A was further purified on a Superdex 75 26/600 pg column in 50 mM Acetate, pH 5.

NMR spectroscopy

^{15}N- Carr-Purcell-Meiboom-Gill (CPMG) data were carried out on a 900 MHz Bruker spectrometer equipped with a cryogenic triple resonance probe. NMR data were collected and processed using TopSpin 3.2. Measurements for BcChi-A were performed at 300 K on a 1 mM ^{13}C, ^{15}N labelled sample in 50 mM Acetate, pH 5.0, while YjbJ experiments were conducted at 298 K on a 1 mM sample of ^{13}C, ^{15}N labelled in 25 mM Na_2PO_4, pH 6.5. ^{15}N- CPMG[32,33] data was acquired as pseudo 3Ds with a fixed total CPMG relaxation period (T_{CPMG}) of 40 ms, and 16 n_{cyc} values ranging from 1 to 40. A reference spectrum was collected with no CPMG relaxation delay (n_{cyc}=0), and duplicate measurements at n_{cyc} of 4 and 20 were collected for error estimation. The effective transverse relaxation rate $R_{2,eff}$ was calculated from these datasets, and errors were taken as the larger of noise-based versus duplicate-based error estimation. Each dataset was collected with 32 scans per FID for the BcChi-A or 8 scans per FID for the YjbJ protein with 2 sec repetitive delay between scans. All CPMG traces for both proteins are in Supplemental Data 1.

Backbone assignments for the BcChi-A and YjbJ were obtained from BMRB accession codes 11441 and 5105, respectively.

NMR data analysis and fitting

bioRxiv preprint doi: https://doi.org/10.1101/2025.03.19.642801; this version posted July 1, 2026. The copyright holder for this preprint (which was not certified by peer review) is the author/funder, who has granted bioRxiv a license to display the preprint in perpetuity. It is made available under a CC-BY 4.0 International license.

Peak heights for individual residues were collected from each 2D ^{15}N-HSQC in the pseudo 3D ^{15}N-CPMG by lineshape fitting using PINT[34] software. The $R_{2,eff}$ at a given CPMG field strength (ν_{cpmg}) was calculated using:

$$R_{2,eff} = \frac{1}{T_{CPMG}} \ln \frac{V(0)}{V(v_{cpmg})}$$

where T_{cpmg} is the constant CPMG relaxation period, V(0) is the peak volume and $V(\nu_{cpmg})$ is the peak volume at each CPMG field strength.

Relaxation dispersion curves, expressed as $R_{2,\ eff}$ versus CPMG field strength, were fit per residue using ChemEx software (https://github.com/gbouvignies/chemex) [35].

1 Kneller, J. M., Lu, M. & Bracken, C. An effective method for the discrimination of motional anisotropy and chemical exchange. *J Am Chem Soc* **124**, 1852-1853 (2002). https://doi.org:10.1021/ja017461k

2 Garcia de la Torre, J., Huertas, M. L. & Carrasco, B. HYDRONMR: prediction of NMR relaxation of globular proteins from atomic-level structures and hydrodynamic calculations. *J Magn Reson* **147**, 138-146 (2000). https://doi.org:10.1006/jmre.2000.2170

3 Bernado, P., Garcia de la Torre, J. & Pons, M. Interpretation of 15N NMR relaxation data of globular proteins using hydrodynamic calculations with HYDRONMR. *J Biomol NMR* **23**, 139-150 (2002). https://doi.org:10.1023/a:1016359412284

4 Raza, T. *et al.* Insights into the NF-κB-DNA Interaction through NMR Spectroscopy. *ACS Omega* **6**, 12877-12886 (2021). https://doi.org:10.1021/acsomega.1c01299

5 Korn, S. M. *et al.* (1)H, (13)C, and (15)N backbone chemical shift assignments of the nucleic acid-binding domain of SARS-CoV-2 non-structural protein 3e. *Biomol NMR Assign* **14**, 329-333 (2020). https://doi.org:10.1007/s12104-020-09971-6

6 Johnson, E. C., Lazar, G. A., Desjarlais, J. R. & Handel, T. M. Solution structure and dynamics of a designed hydrophobic core variant of ubiquitin. *Structure* **7**, 967-976 (1999). https://doi.org:https://doi.org/10.1016/S0969-2126(99)80123-3

7 Pei, J. & Grishin, N. V. AL2CO: calculation of positional conservation in a protein sequence alignment. *Bioinformatics* **17**, 700-712 (2001). https://doi.org:10.1093/bioinformatics/17.8.700

bioRxiv preprint doi: https://doi.org/10.1101/2025.03.19.642801; this version posted July 1, 2026. The copyright holder for this preprint (which was not certified by peer review) is the author/funder, who has granted bioRxiv a license to display the preprint in perpetuity. It is made available under a CC-BY 4.0 International license.

8 Shrake, A. & Rupley, J. A. Environment and exposure to solvent of protein atoms. Lysozyme and insulin. *J Mol Biol* **79**, 351-371 (1973). https://doi.org:10.1016/0022-2836(73)90011-9
9 McGibbon, R. T. *et al.* MDTraj: A Modern Open Library for the Analysis of Molecular Dynamics Trajectories. *Biophys J* **109**, 1528-1532 (2015). https://doi.org:10.1016/j.bpj.2015.08.015
10 Chen, H. & Zhou, H. X. Prediction of solvent accessibility and sites of deleterious mutations from protein sequence. *Nucleic Acids Res* **33**, 3193-3199 (2005). https://doi.org:10.1093/nar/gki633
11 Hoch, J. C. *et al.* Biological Magnetic Resonance Data Bank. *Nucleic Acids Res* **51**, D368-D376 (2023). https://doi.org:10.1093/nar/gkac1050
12 Lin, Z. *et al.* Evolutionary-scale prediction of atomic-level protein structure with a language model. *Science* **379**, 1123-1130 (2023). https://doi.org:10.1126/science.ade2574
13 Hauser, M., Steinegger, M. & Soding, J. MMseqs software suite for fast and deep clustering and searching of large protein sequence sets. *Bioinformatics* **32**, 1323-1330 (2016). https://doi.org:10.1093/bioinformatics/btw006
14 Henzler-Wildman, K. A. *et al.* Intrinsic motions along an enzymatic reaction trajectory. *Nature* **450**, 838-844 (2007). https://doi.org:10.1038/nature06410
15 Elings, W. *et al.* Two beta-Lactamase Variants with Reduced Clavulanic Acid Inhibition Display Different Millisecond Dynamics. *Antimicrob Agents Chemother* **65**, e0262820 (2021). https://doi.org:10.1128/AAC.02628-20
16 Paukovich, N. *et al.* Biliverdin Reductase B Dynamics Are Coupled to Coenzyme Binding. *J Mol Biol* **430**, 3234-3250 (2018). https://doi.org:10.1016/j.jmb.2018.06.015
17 Eisenmesser, E. Z., Bosco, D. A., Akke, M. & Kern, D. Enzyme dynamics during catalysis. *Science* **295**, 1520-1523 (2002). https://doi.org:10.1126/science.1066176
18 Beaumont, V. A. *Functional Implications of Conformational Dynamics of Protein Tyrosine Phosphatases*, Yale University, (2019).
19 Hansen, A. L., Xiang, X., Yuan, C., Bruschweiler-Li, L. & Bruschweiler, R. Excited-state observation of active K-Ras reveals differential structural dynamics of wild-type versus oncogenic G12D and G12C mutants. *Nat Struct Mol Biol* **30**, 1446-1455 (2023). https://doi.org:10.1038/s41594-023-01070-z
20 McDonald, L. R., Boyer, J. A. & Lee, A. L. Segmental motions, not a two-state concerted switch, underlie allostery in CheY. *Structure* **20**, 1363-1373 (2012). https://doi.org:10.1016/j.str.2012.05.008
21 Aspholm, E. E., Lidman, J. & Burmann, B. M. Structural basis of substrate recognition and allosteric activation of the proapoptotic mitochondrial HtrA2 protease. *Nat Commun* **15**, 4592 (2024). https://doi.org:10.1038/s41467-024-48997-5
22 Beach, H., Cole, R., Gill, M. L. & Loria, J. P. Conservation of mus-ms enzyme motions in the apo- and substrate-mimicked state. *J Am Chem Soc* **127**, 9167-9176 (2005). https://doi.org:10.1021/ja0514949
23 Davulcu, O., Flynn, P. F., Chapman, M. S. & Skalicky, J. J. Intrinsic domain and loop dynamics commensurate with catalytic turnover in an induced-fit enzyme. *Structure* **17**, 1356-1367 (2009). https://doi.org:10.1016/j.str.2009.08.014

bioRxiv preprint doi: https://doi.org/10.1101/2025.03.19.642801; this version posted July 1, 2026. The copyright holder for this preprint (which was not certified by peer review) is the author/funder, who has granted bioRxiv a license to display the preprint in perpetuity. It is made available under a CC-BY 4.0 International license.

24 Zhang, Y. & Skolnick, J. TM-align: a protein structure alignment algorithm based on the TM-score. *Nucleic Acids Res* **33**, 2302-2309 (2005). https://doi.org:10.1093/nar/gki524

25 Case, D. A. Normal mode analysis of protein dynamics. *Current Opinion in Structural Biology* **4**, 285-290 (1994). https://doi.org:https://doi.org/10.1016/S0959-440X(94)90321-2

26 Zhang, S. *et al.* ProDy 2.0: increased scale and scope after 10 years of protein dynamics modelling with Python. *Bioinformatics* **37**, 3657-3659 (2021). https://doi.org:10.1093/bioinformatics/btab187

27 Loshchilov, I. Decoupled weight decay regularization. *arXiv preprint arXiv:1711.05101* (2017).

28 Menon, A. K. *et al.* Long-tail learning via logit adjustment. *arXiv preprint arXiv:2007.07314* (2020).

29 Waskom, M. L. Seaborn: statistical data visualization. *Journal of Open Source Software* **6**, 3021 (2021).

30 Yee, A. *et al.* An NMR approach to structural proteomics. *Proc Natl Acad Sci U S A* **99**, 1825-1830 (2002). https://doi.org:10.1073/pnas.042684599

31 Taira, T. *et al.* Cloning and characterization of a small family 19 chitinase from moss (Bryum coronatum). *Glycobiology* **21**, 644-654 (2011). https://doi.org:10.1093/glycob/cwq212

32 Hansen, D. F., Vallurupalli, P. & Kay, L. E. An improved 15N relaxation dispersion experiment for the measurement of millisecond time-scale dynamics in proteins. *J Phys Chem B* **112**, 5898-5904 (2008). https://doi.org:10.1021/jp074793o

33 Jiang, B., Yu, B., Zhang, X., Liu, M. & Yang, D. A (15)N CPMG relaxation dispersion experiment more resistant to resonance offset and pulse imperfection. *J Magn Reson* **257**, 1-7 (2015). https://doi.org:10.1016/j.jmr.2015.05.003

34 Ahlner, A., Carlsson, M., Jonsson, B. H. & Lundstrom, P. PINT: a software for integration of peak volumes and extraction of relaxation rates. *J Biomol NMR* **56**, 191-202 (2013). https://doi.org:10.1007/s10858-013-9737-7

35 Vallurupalli, P., Bouvignies, G. & Kay, L. E. Studying "invisible" excited protein states in slow exchange with a major state conformation. *J Am Chem Soc* **134**, 8148-8161 (2012). https://doi.org:10.1021/ja3001419

bioRxiv preprint doi: https://doi.org/10.1101/2025.03.19.642801; this version posted July 1, 2026. The copyright holder for this preprint (which was not certified by peer review) is the author/funder, who has granted bioRxiv a license to display the preprint in perpetuity. It is made available under a CC-BY 4.0 International license.

bioRxiv preprint doi: https://doi.org/10.1101/2025.03.19.642801; this version posted July 1, 2026. The copyright holder for this preprint (which was not certified by peer review) is the author/funder, who has granted bioRxiv a license to display the preprint in perpetuity. It is made available under a CC-BY 4.0 International license.

Extended Data

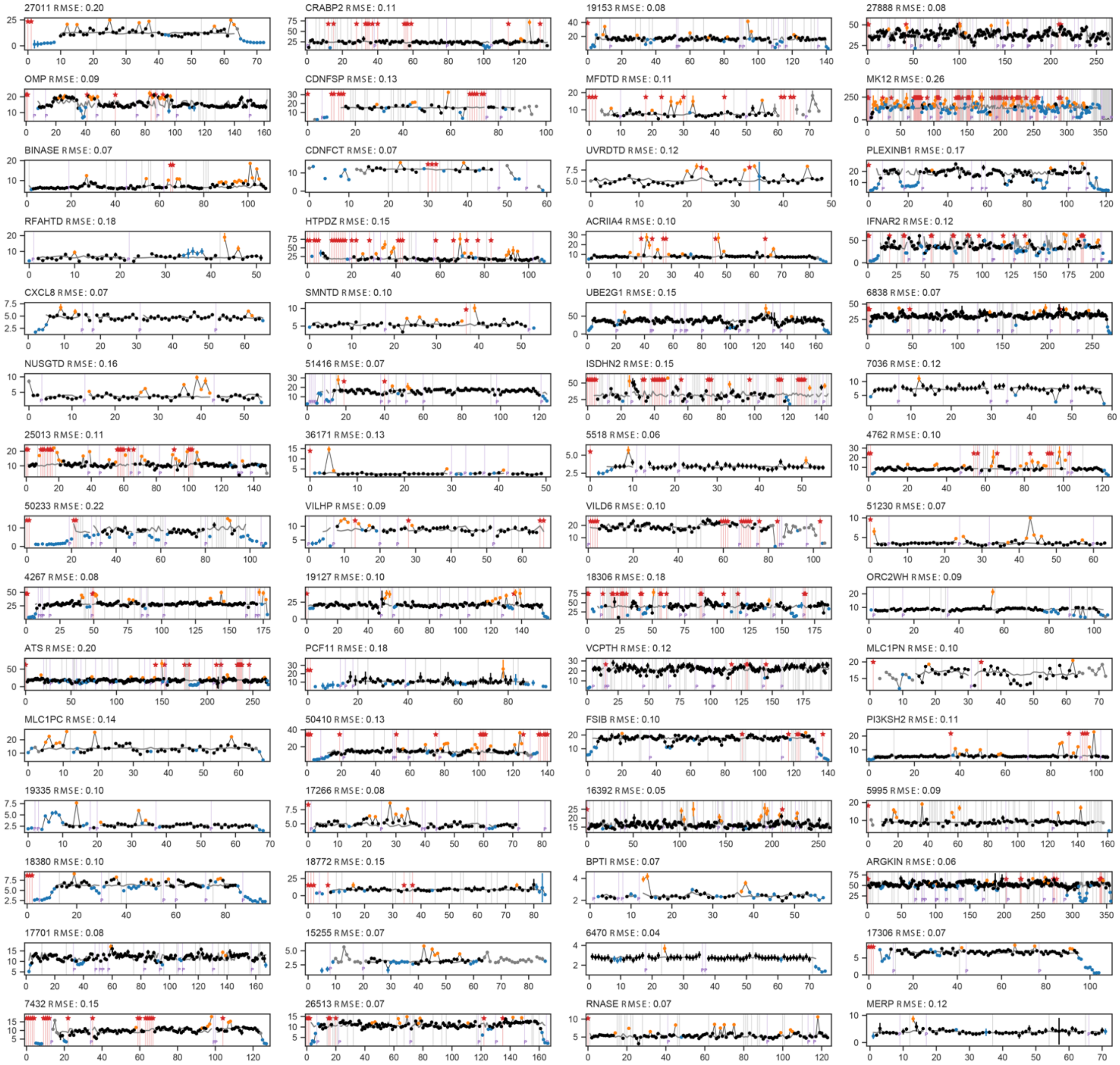

Extended Data Figure 1: (page 1/3) ^{15}N R_2/R_1 data and labels for all 133 proteins in RelaxDB and the 30 which were initially curated but excluded from final evaluation set for various reasons (see Methods). Calculated R_2/R_1 from HYDRONMR[25,26] for rigid tumbling is depicted by grey line. Colors used are the same as in Fig. 1. Title for each is RelaxDB entry ID followed by normalized RMSD.

bioRxiv preprint doi: https://doi.org/10.1101/2025.03.19.642801; this version posted July 1, 2026. The copyright holder for this preprint (which was not certified by peer review) is the author/funder, who has granted bioRxiv a license to display the preprint in perpetuity. It is made available under a CC-BY 4.0 International license.

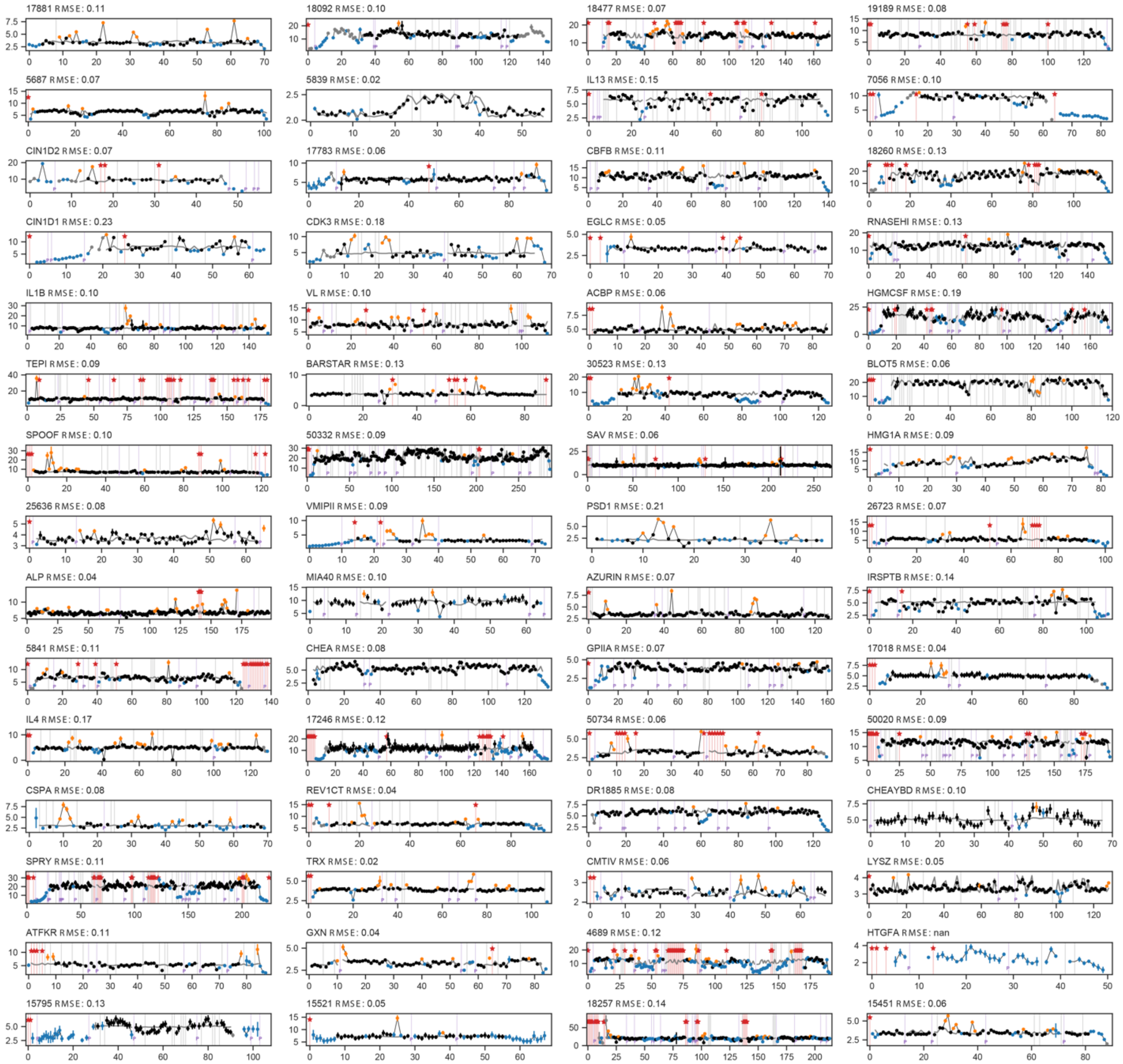

Extended Data Figure 1: (page 2/3)

bioRxiv preprint doi: https://doi.org/10.1101/2025.03.19.642801; this version posted July 1, 2026. The copyright holder for this preprint (which was not certified by peer review) is the author/funder, who has granted bioRxiv a license to display the preprint in perpetuity. It is made available under a CC-BY 4.0 International license.

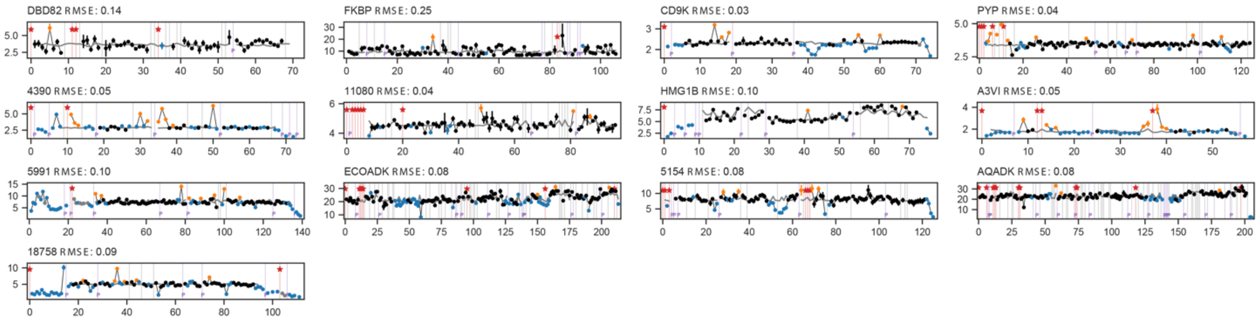

Initially curated, but excluded

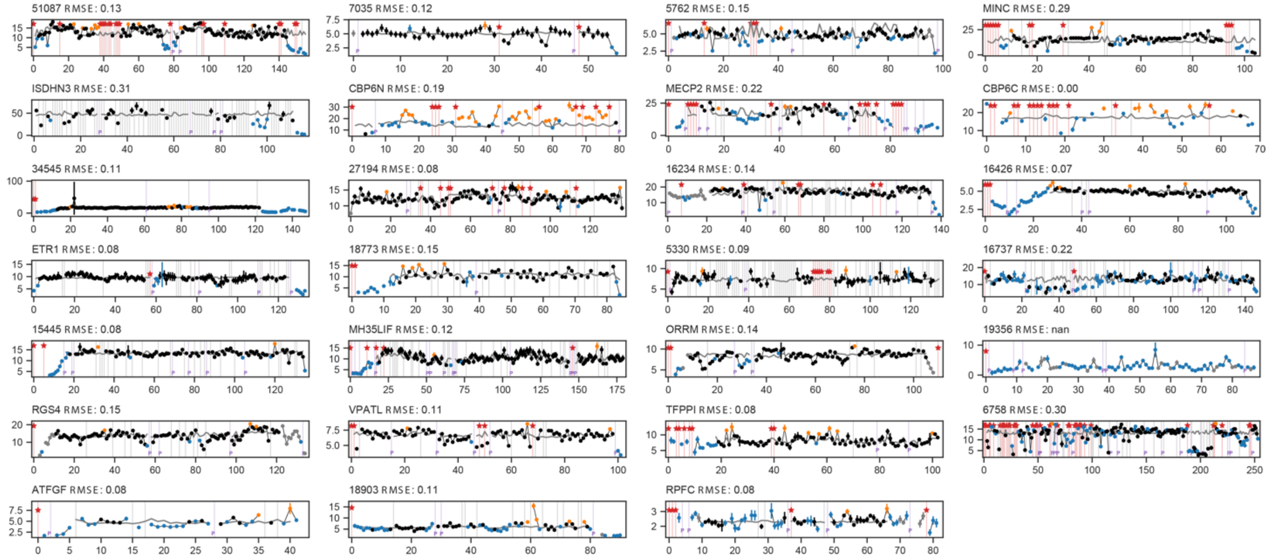

Extended Data Figure 1: (page 3/3)

bioRxiv preprint doi: https://doi.org/10.1101/2025.03.19.642801; this version posted July 1, 2026. The copyright holder for this preprint (which was not certified by peer review) is the author/funder, who has granted bioRxiv a license to display the preprint in perpetuity. It is made available under a CC-BY 4.0 International license.

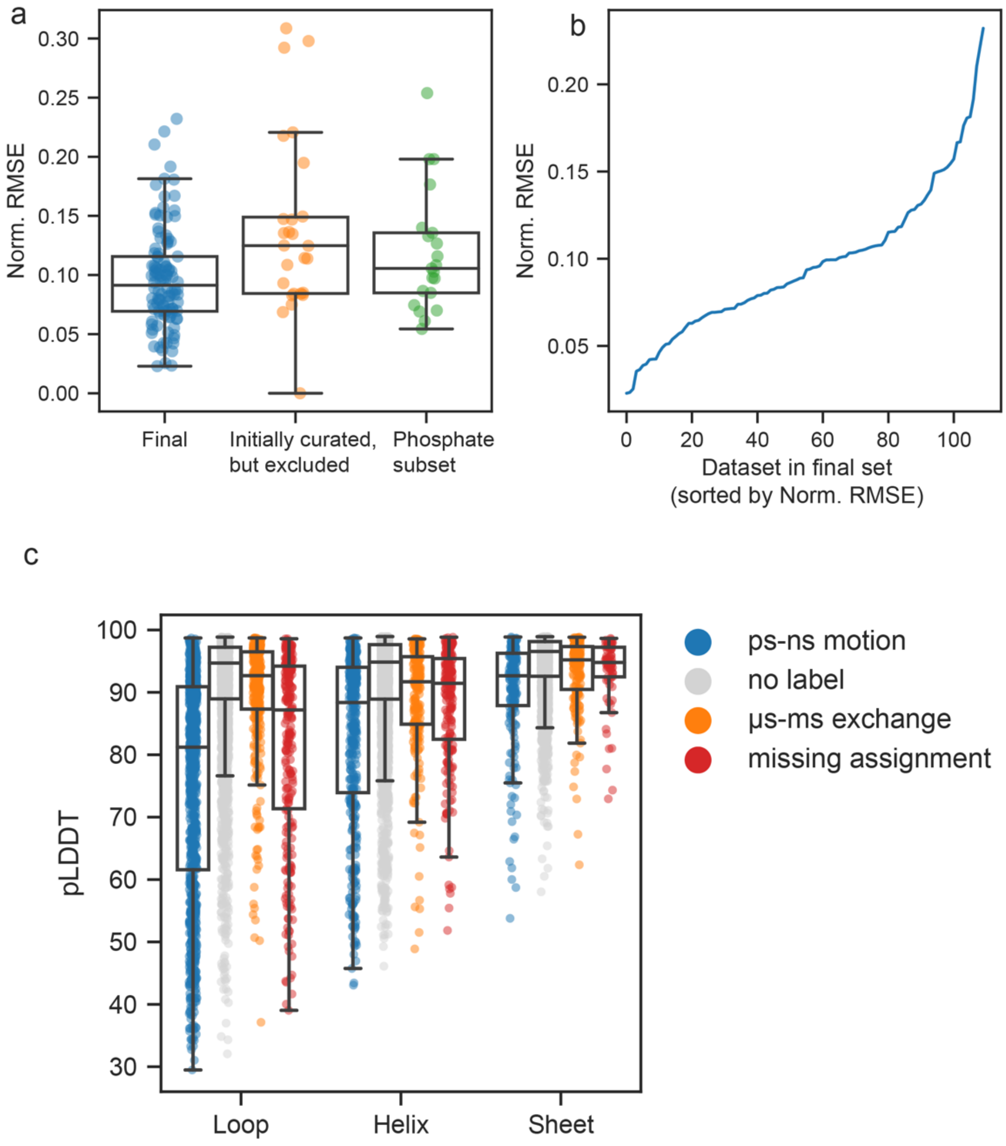

Extended Data Figure 2: (a) RMSE normalized to median R_2/R_1 per protein for the final RelaxDB dataset, protein datasets that were initially curated but excluded, and proteins with biological function involving phosphate binding, but measured in phosphate buffer. n=160 proteins. (b) Norm. RMSE distribution of final RelaxDB dataset used for evaluation. (c) pLDDT (predicted local distance difference test) from AF2 by secondary structure and dynamics type. Low AF2 pLDDT is most indicative of ps-ns motion in RelaxDB. Box plots depict median and 25/75% interquartile range, whiskers = 1.5 *interquartile range, n=12,584 residues.

bioRxiv preprint doi: https://doi.org/10.1101/2025.03.19.642801; this version posted July 1, 2026. The copyright holder for this preprint (which was not certified by peer review) is the author/funder, who has granted bioRxiv a license to display the preprint in perpetuity. It is made available under a CC-BY 4.0 International license.

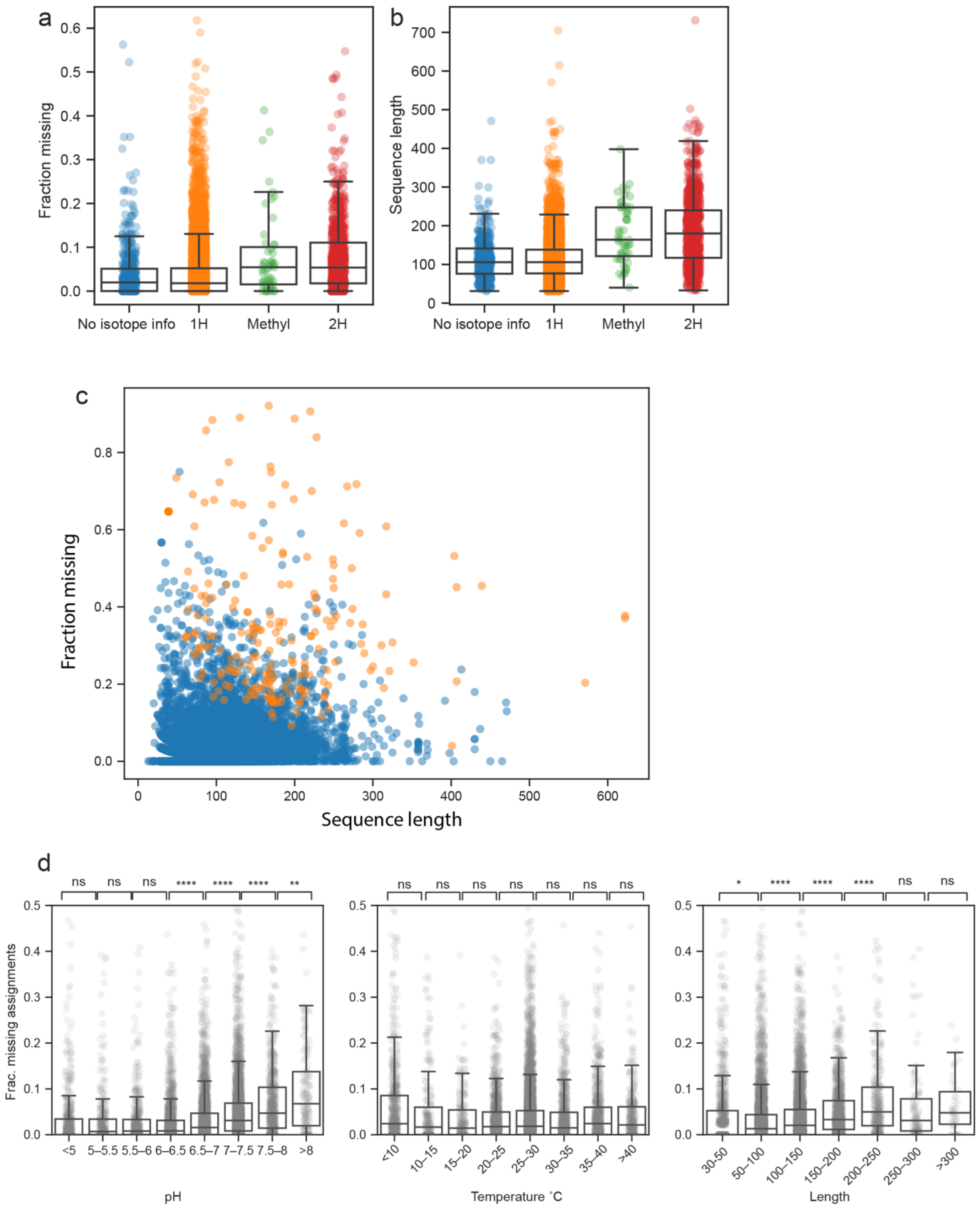

bioRxiv preprint doi: https://doi.org/10.1101/2025.03.19.642801; this version posted July 1, 2026. The copyright holder for this preprint (which was not certified by peer review) is the author/funder, who has granted bioRxiv a license to display the preprint in perpetuity. It is made available under a CC-BY 4.0 International license.

Extended Data Figure 3: Statistics from curating the "missing assignment BMRB" (mBMRB). We removed proteins with deuteration or methyl or specific labelling, based on metadata. Some proteins had no isotope information, yet we elected to keep them, as they had fewer missing assignments compared to proteins with ^{1}H isotope labels and were overall shorter than other isotope categories. (a) Fraction missing split by isotope category (n=11,268 proteins). (b) Sequence length by isotope category (n=11,268 proteins). (c) We removed entries with 15 or more consecutive missing assignments (orange). (d) Missing assignments as a function of pH, Temperature, and sequence length. In (a,b,d): Box plots depict median and 25/75% interquartile range, whiskers = 1.5 times the interquartile range. In d: statistical comparisons by two-tailed independent-samples t-test with multiple comparisons adjustment. ns: 0.05 < p <= 1. *: 0.01 < p <= 0.05. **: 0.001 < p <= 0.01. ***: 0.0001 < p <= 0.001. ****: p <= 0.0001.

bioRxiv preprint doi: https://doi.org/10.1101/2025.03.19.642801; this version posted July 1, 2026. The copyright holder for this preprint (which was not certified by peer review) is the author/funder, who has granted bioRxiv a license to display the preprint in perpetuity. It is made available under a CC-BY 4.0 International license.

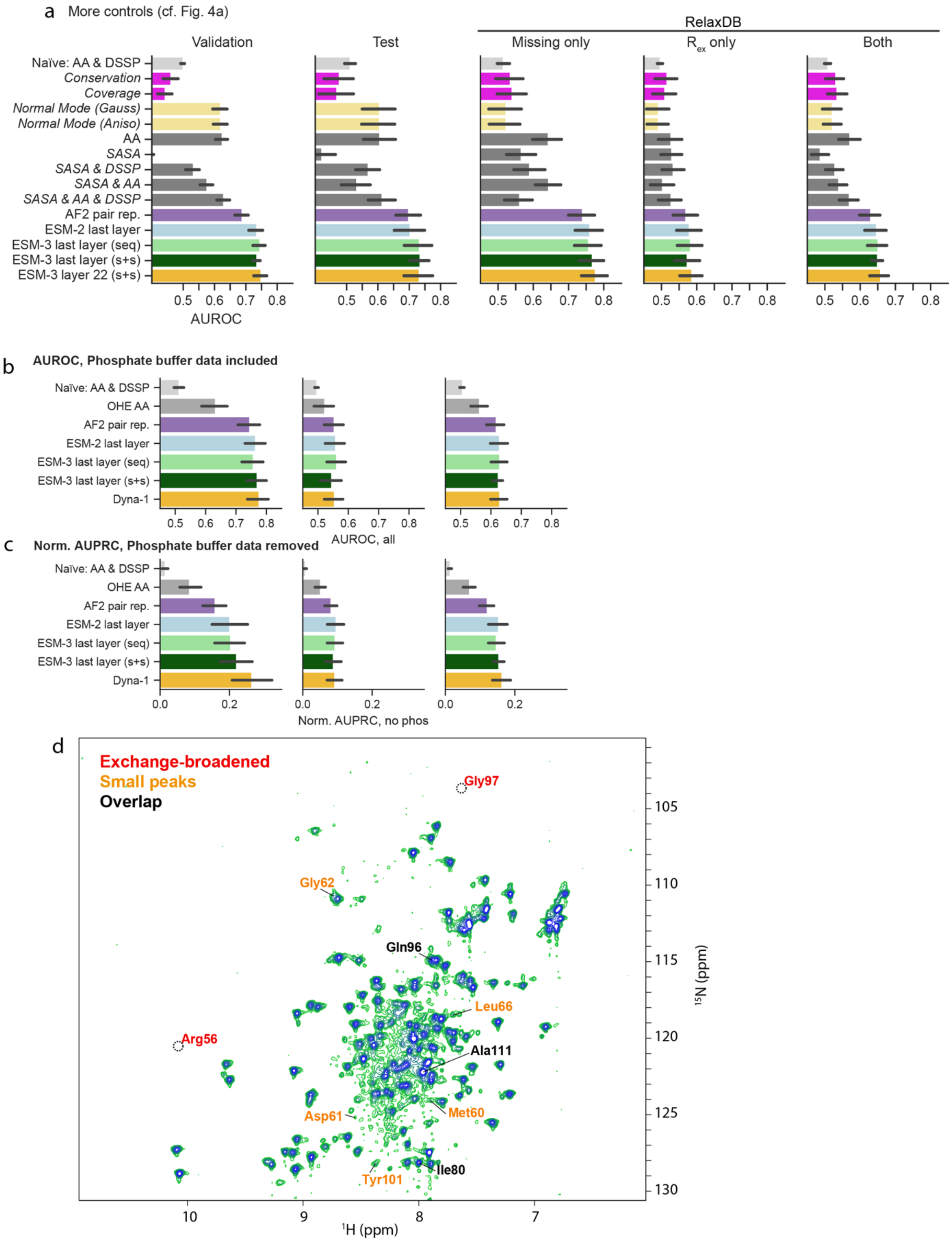

Extended Data Figure 4. (a) Validation, test, and RelaxDB datasets comparing further benchmarks at training cutoff of 30% sequence identity, TMscore 0.5. Benchmark models in italics are not in Fig. 4a. (validation: n=500 proteins, test: n=100 proteins, RelaxDB: n=112 proteins). (b) RelaxDB evaluated by

bioRxiv preprint doi: https://doi.org/10.1101/2025.03.19.642801; this version posted July 1, 2026. The copyright holder for this preprint (which was not certified by peer review) is the author/funder, who has granted bioRxiv a license to display the preprint in perpetuity. It is made available under a CC-BY 4.0 International license.

AUROC across different models tested, using all 133 proteins including those with exchange from phosphate binding (cf. **Fig. 4a**). (c) RelaxDB evaluated across different models evaluated by normalized AUPRC, with phosphate binding proteins removed (n=112 proteins). In (a,b,c), bars represent mean, error bars represent 95% confidence interval evaluated over proteins in RelaxDB. (d) ^{1}H-^{15}N HSQC of NtrC at 25Ċ, annotated with residues for which assignments exist in BMRB entry 4527 (non-phosphorylated NtrC), but relaxation data is missing from BMRB entry 4762. The Arg56 and Gly97 assignments in entry 4762 (shown in red here) are presumed to be erroneously copied from entry 4528, which are assignments corresponding to phosphorylated NtrC. All residues labeled orange are very weak peaks indicative of R_{ex}. For these, we concluded exchange broadening was the reason for no reported relaxation data. Black residues are overlapped peaks.

bioRxiv preprint doi: https://doi.org/10.1101/2025.03.19.642801; this version posted July 1, 2026. The copyright holder for this preprint (which was not certified by peer review) is the author/funder, who has granted bioRxiv a license to display the preprint in perpetuity. It is made available under a CC-BY 4.0 International license.

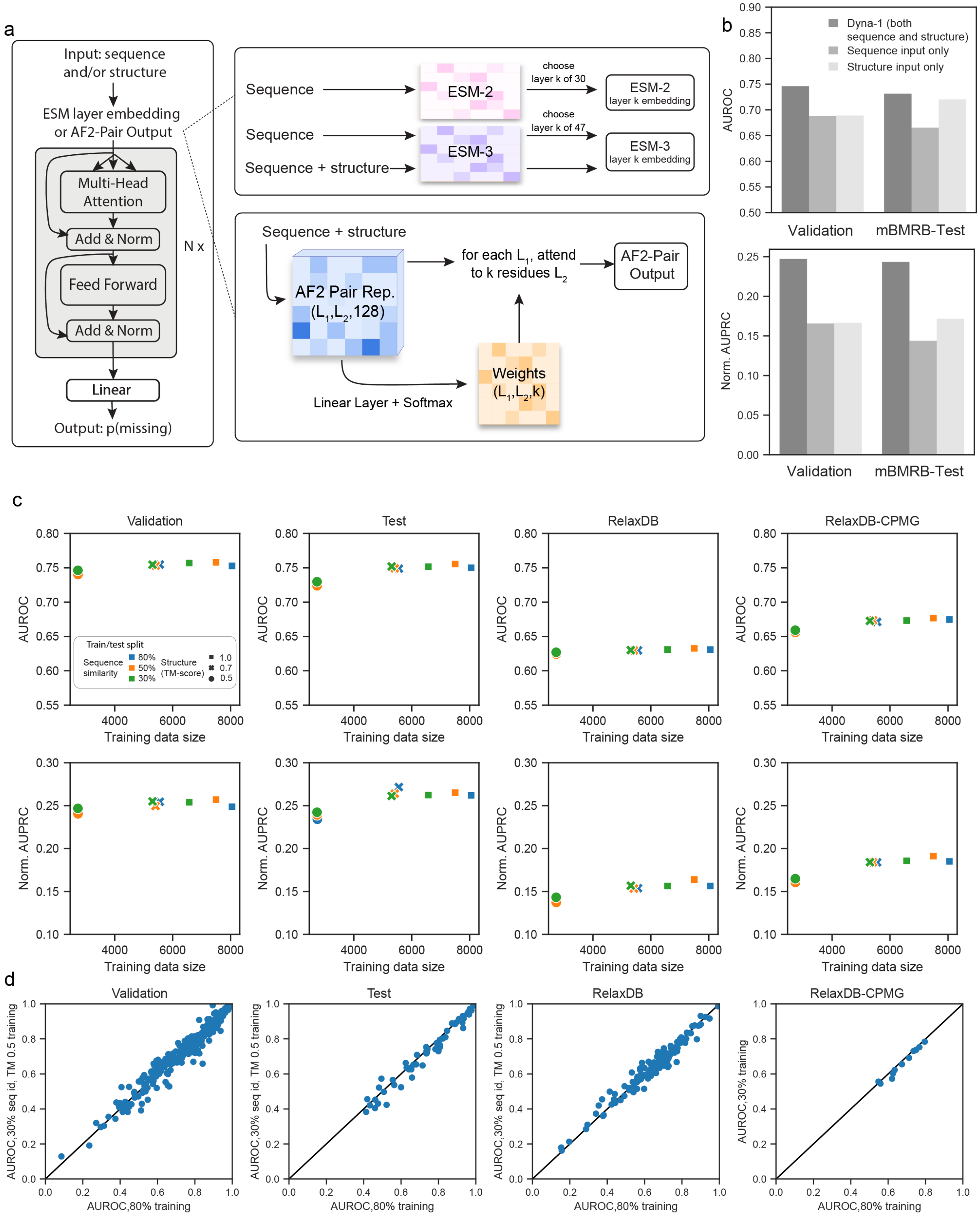

Extended Data Figure 5: Details of training Dyna-1. (a) Architectures of tested ESM and AF2 inputs, outlining the model architecture. At training time, either a sequence-structure pair is passed through AF2 or ESM-3, or a sequence is passed through ESM-2 or ESM-3. The corresponding embedding from the

bioRxiv preprint doi: https://doi.org/10.1101/2025.03.19.642801; this version posted July 1, 2026. The copyright holder for this preprint (which was not certified by peer review) is the author/funder, who has granted bioRxiv a license to display the preprint in perpetuity. It is made available under a CC-BY 4.0 International license.

k^th^ layer from ESM or AF2-pair output is fixed ("frozen") and used as input into a one-dimensional Transformer architecture. Output is one value per residue, the logit for that residue being missing. (b) Comparing Dyna-1 performance given both sequence and structure (blue) vs. sequence only (orange) or structure only (green). Performance is best when given both sequence and structure. (c) Dyna-1 performance given different training split cutoffs based on sequence identity and structure similarity (cf. Fig. 3d) across all datasets. (d) AUROC per protein for either training with the 80% sequence identity and 1.0 TM-score split or 30% sequence identity and 0.5 TM-score split. Performance on individual proteins in the validation, test, RelaxDB, and RelaxDB-CPMG does not significantly change between more stringent sequence similarity cutoffs.

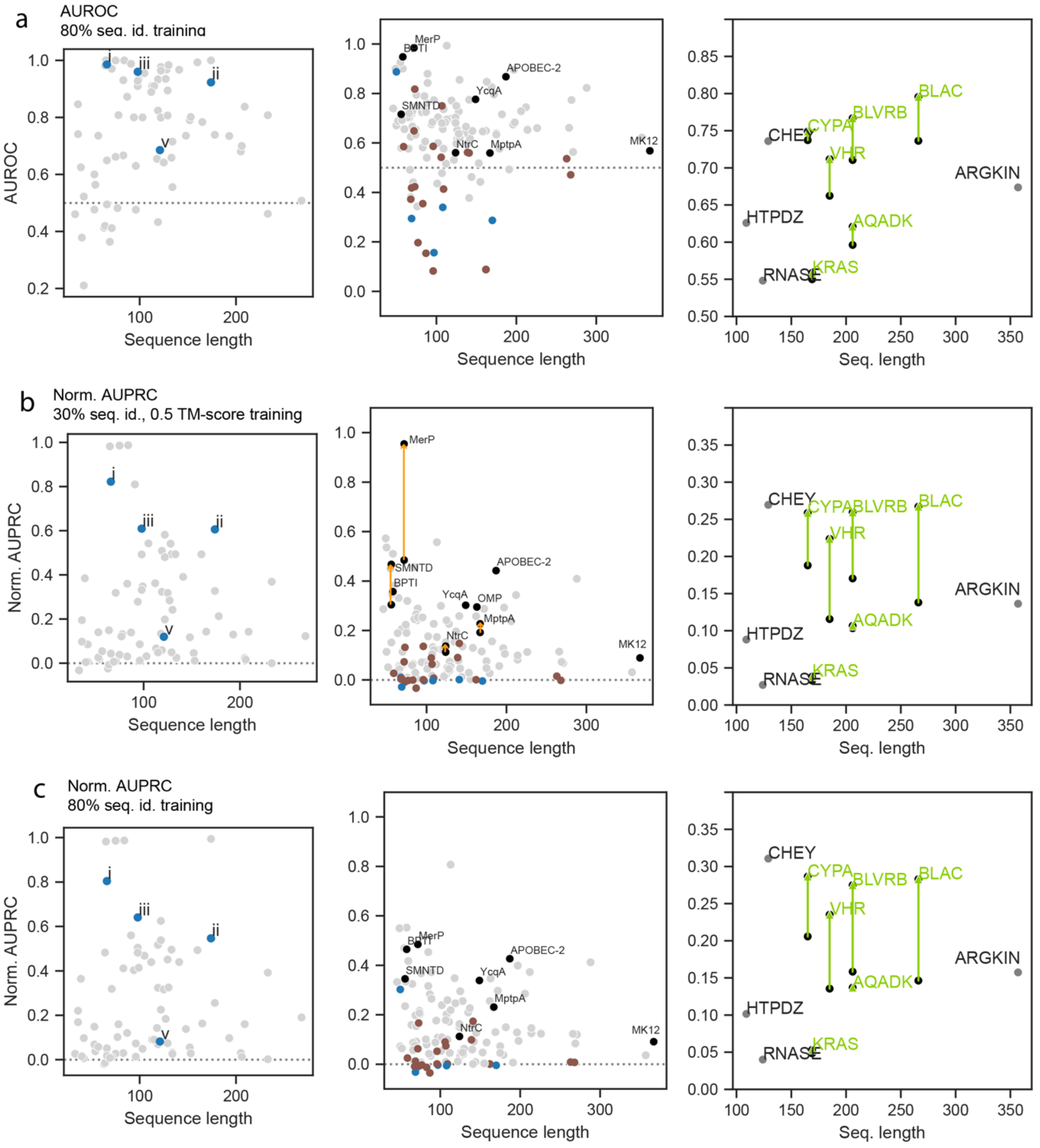

bioRxiv preprint doi: https://doi.org/10.1101/2025.03.19.642801; this version posted July 1, 2026. The copyright holder for this preprint (which was not certified by peer review) is the author/funder, who has granted bioRxiv a license to display the preprint in perpetuity. It is made available under a CC-BY 4.0 International license.

Extended Data Figure 6. Dyna-1 trained with 80% sequence identity cutoff is comparable to results presented in main text for 30% sequence identity and 0.5 TM-score cutoff. (a) AUROC for mBMRB-Test (cf. Fig. 3e), RelaxDB (cf. Fig. 4b), RelaxDB-CPMG (cf. Fig. 5d). Coloring for each follows respective main-text figures. (b) Normalized AUPRC per protein for Dyna-1, trained with 30% sequence identity and 0.5 TM-score cutoff. (c) Normalized AUPRC per protein for Dyna-1, trained with 80% sequence identity cutoff.

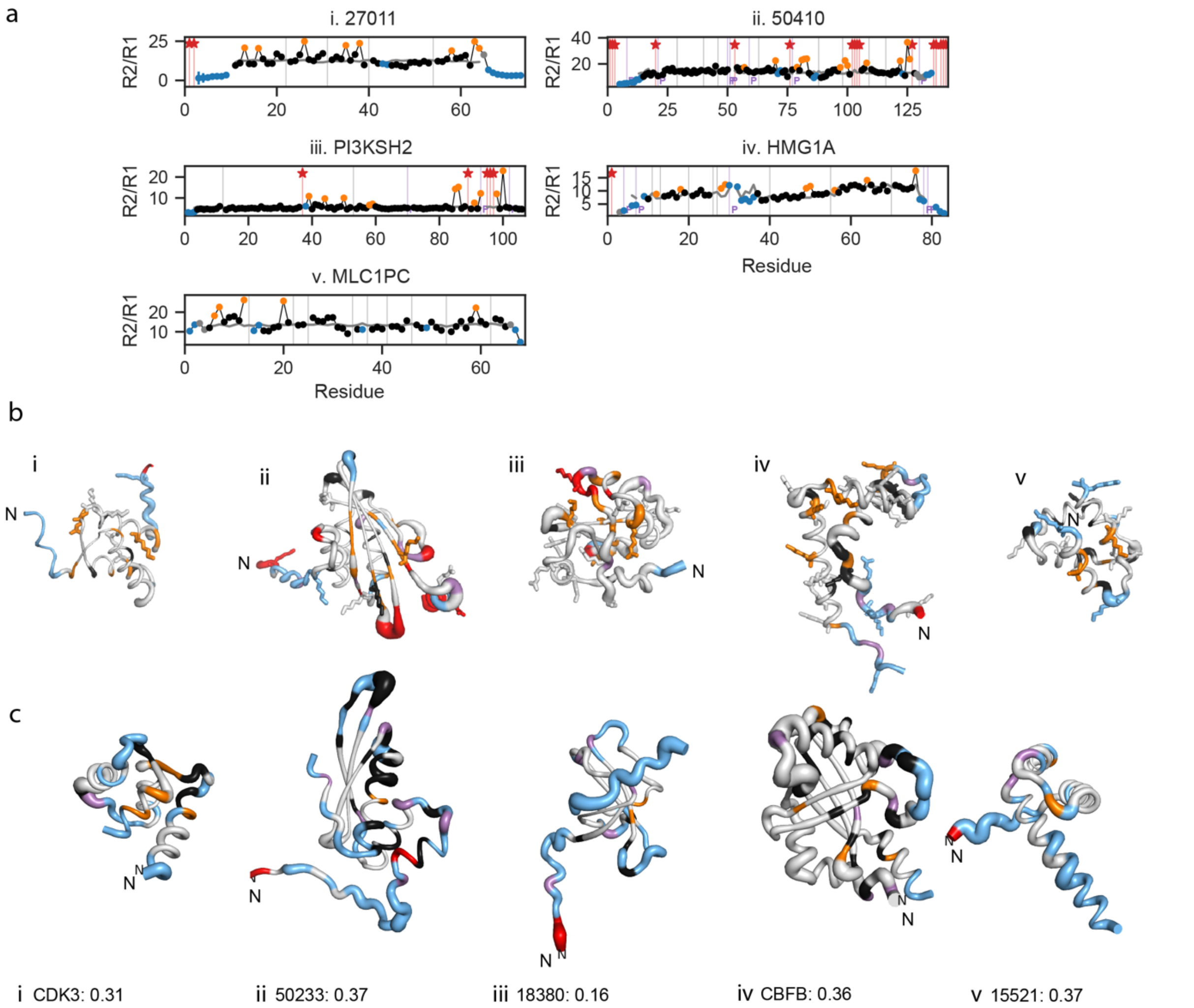

Extended Data Figure 7: Some proteins with poorly predicted exchange in RelaxDB reflect phosphate-binding exchange or high probability for ps-ns motion. Numerous proteins in RelaxDB predicted with an AUROC < 0.5 are these proteins that bind phosphate ion moieties during their biological function, yet the NMR experiments were measured in phosphate buffer. (a) R_2/R_1 data for 6 of these proteins which bind RNA or DNA. (b) All positively charged sidechains (arginine, lysine) are depicted in stick form. Many of the residues with experimental exchange (orange) that are not predicted by Dyna-1 are on the surface of these proteins and can be explained by phosphate binding. (c) Proteins with low AUROC and high p(exchange) predicted in disordered N- or C- termini, or areas with characterized ps-ns motion, labeled in blue in Fig. 4b and here. In (b,c): structures are sized by Dyna-1 p(exchange) and colored by experimental labels as in Fig. 4.

bioRxiv preprint doi: https://doi.org/10.1101/2025.03.19.642801; this version posted July 1, 2026. The copyright holder for this preprint (which was not certified by peer review) is the author/funder, who has granted bioRxiv a license to display the preprint in perpetuity. It is made available under a CC-BY 4.0 International license.

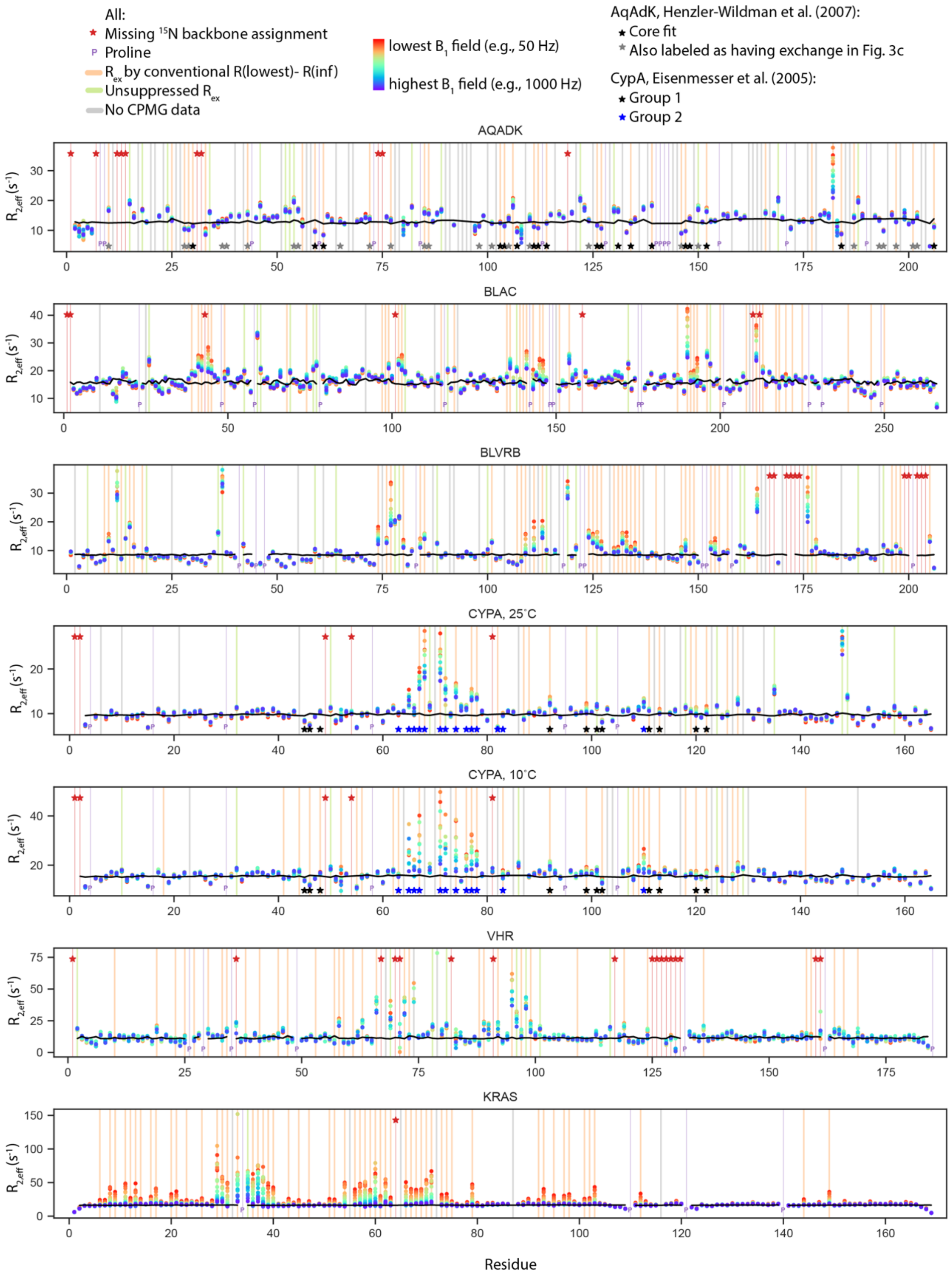

Extended Data Figure 8: RelaxDB-CPMG datasets where dispersion data was available for all residues. Legend: Missing assignments (red star), prolines (purple "P"), exchange via R_{ex} calculated conventionally (light orange), residues with unsuppressed R_{ex} (light green), residues with no CPMG data due to peak

bioRxiv preprint doi: https://doi.org/10.1101/2025.03.19.642801; this version posted July 1, 2026. The copyright holder for this preprint (which was not certified by peer review) is the author/funder, who has granted bioRxiv a license to display the preprint in perpetuity. It is made available under a CC-BY 4.0 International license.

overlap (gray). The color gradient represents B_1 field strength from lowest (red, e.g., 50 Hz) to highest (purple, e.g., 1000 Hz). The AqAdk and CypA datasets are annotated with residue groups reported in refs. [41] and [38], respectively.

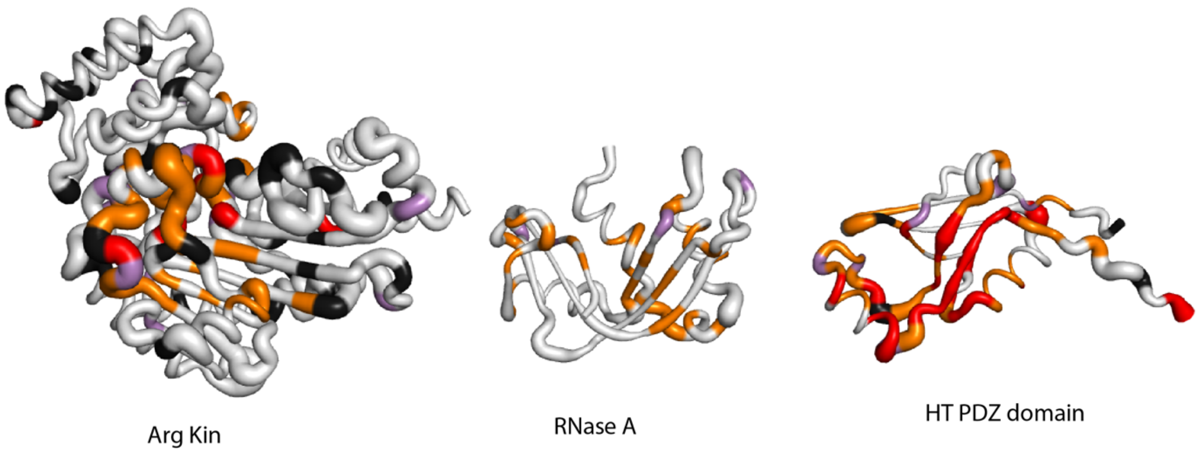

Extended Data Figure 9: Additional ^{15}N CPMG datasets with no dispersion data published for all residues (cf. Figure 5e).
